# *Drosophila* germ granules are structured and contain homotypic mRNA clusters

**DOI:** 10.1038/ncomms8962

**Published:** 2015-08-05

**Authors:** Tatjana Trcek, Markus Grosch, Andrew York, Hari Shroff, Timothée Lionnet, Ruth Lehmann

**Affiliations:** 1 Department of Cell Biology, HHMI, Skirball Institute of Biomolecular Medicine, NYU School of Medicine, 540 First Avenue, New York, New York 10016, USA; 2 Section on High Resolution Optical Imaging, National Institute of Biomedical Imaging and Bioengineering, NIH, BG 13 RM G800, 13 South Dr, Bethesda, Maryland 20814, USA; 3 Transcription Imaging Consortium, Janelia Research Campus, Howard Hughes Medical Institute, 19700 Helix Drive, Ashburn, Virginia 20147, USA

## Abstract

Germ granules, specialized ribonucleoprotein particles, are a hallmark of all germ cells. In *Drosophila*, an estimated 200 mRNAs are enriched in the germ plasm, and some of these have important, often conserved roles in germ cell formation, specification, survival and migration. How mRNAs are spatially distributed within a germ granule and whether their position defines functional properties is unclear. Here we show, using single-molecule FISH and structured illumination microscopy, a super-resolution approach, that mRNAs are spatially organized within the granule whereas core germ plasm proteins are distributed evenly throughout the granule. Multiple copies of single mRNAs organize into ‘homotypic clusters' that occupy defined positions within the center or periphery of the granule. This organization, which is maintained during embryogenesis and independent of the translational or degradation activity of mRNAs, reveals new regulatory mechanisms for germ plasm mRNAs that may be applicable to other mRNA granules.

## Abstract (web summary)

What regulates mRNAs transcript localization in the germ granules in *Drosophila* is unclear. Here Trcek *et al.* identify that germ plasm proteins are homogeneously distributed in germ granules but once localized, individual mRNAs form homotypic clusters, contributing structure to the germ granules.

## Graphical abstract

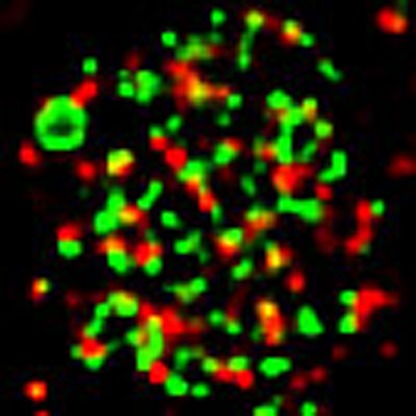

The germ line lineage is critical for the reproductive success of any species. Characteristic to all germ cells are specialized membraneless, ribonucleoprotein granules in the form of maternally inherited germ plasm or nuclear associated nuage. Among organisms, germ granules differ depending on the timing of their formation. In species like *Drosophila melanogaster*, these granules are synthesized during oogenesis and accumulate in the germ plasm in the egg and early embryo, while in *Caenorhabditis elegans* they form during germ cell specification, and in other species, like mouse or human, they are only found later during germ cell development in the nuage. While the role of germ plasm as a cytoplasmic determinant of germ cell fate was postulated more than 100 years ago, the exact composition of germ line granules is not known^1^,^2^. Genetic analysis in *Drosophila melanogaster* identified highly conserved proteins that are common to all germ granules and critical for their assembly *in vivo*^3^. How these specific factors interact with each other and other protein and mRNA components of the germ granules and how they exert their function on germ cell biology is subject of intense study.

In *Drosophila*, germ plasm is synthesized during oogenesis and assembles at the posterior pole of the egg cell. Initially, the fertilized fly embryo is syncytial and nuclei divide in the center of the developing embryo. Once the nuclei begin migrating towards the surface of the embryo, those that become engulfed by the germ plasm at the posterior will develop into primordial germ cells (PGCs), while the rest will differentiate and give rise to all somatic tissues. Thus, the role of the germ plasm is not only to specify the position of PGC formation but also to prevent differentiation of PGCs into somatic cells, thereby maintaining their totipotency.

On a protein level, *Drosophila* germ plasm is composed of the core germ plasm proteins, Oskar, Vasa and Tudor and a number of proteins involved in various aspects of RNA biology^1^,^3^. Germ plasm formation begins with microtubule-dependent localization of *osk* mRNA to the posterior pole during early oogenesis. Upon localization, *osk* becomes translationally competent, produces Oskar protein, which later recruits Vasa protein along with other germ plasm proteins as well an estimated 200 maternally provided mRNAs, such as *cyclinB* (*cycB*), *nanos* (*nos*), *polar granule component* (*pgc*) and *germ cell less* (*gcl*)^4^. EM revealed that the germ plasm is organized into large, up to 500 nm big granules enriched with polysomes, indicating that germ granules are a site of dynamic translational activity^1^.

mRNA localization to the germ granules occurs during late oogenesis during nurse cell dumping when 15 nurse cells empty their cellular content into a transcriptionally silent oocyte. Using live cell imaging and genetic flourescent-tagging of the mRNA it has been shown that *nos* mRNA localization to the germ plasm occurs passively via a diffusion and entrapment mechanism that is further facilitated by cytoplasmic streaming, which swirls the deposited nurse cell cytoplasm in the oocyte^5^. The efficacy of this localization process is low, accounting for only 4% of deposited *nos* mRNAs^6^. It is believed that other maternally provided mRNAs enriched at the posterior are localized by this process.

Localized mRNAs have important, often conserved roles in germ cell formation, specification, survival and migration^3^. Only localized mRNAs are translationally active, while their unlocalized counterparts, distributed throughout the egg are translationally silent^7^,^8^. Interestingly, to date no instructive, germ cell specific transcriptional ‘master regulator' has been identified indicating that germ line specification and maintenance relies largely, if not entirely on post-transcriptional events, such as mRNA localization. Despite the fact that mRNA localization seems to play a key role in the establishment of the germ line, little is known about how localization organizes transcripts within the germ granules and whether this organization specifies their functional properties. To address this question we used single-mRNA fluorescent *in situ* hybridization (FISH) in combination with structural illumination microscopy, a super-resolution approach, to gain a high-resolution view of the mRNA-bound germ granule. By doing this we determined that the germ plasm proteins are homogeneously distributed within the germ granules while localized mRNAs assume specific positions within the granule. Once localized, multiple copies of an individual mRNA co-organize into homotypic clusters within the same granule thus giving germ granule structure. This organization is maintained during embryogenesis and is also independent of the translational or degradation activity of mRNAs. Our quantitative imaging approach reveals novel regulatory mechanisms of mRNA localization that may be applicable to other mRNA granules and may serve as a platform for studying mRNA localization in other organisms and tissues.

## Results

### Germ plasm proteins are uniformly distributed in the granule

**Figure 1:**
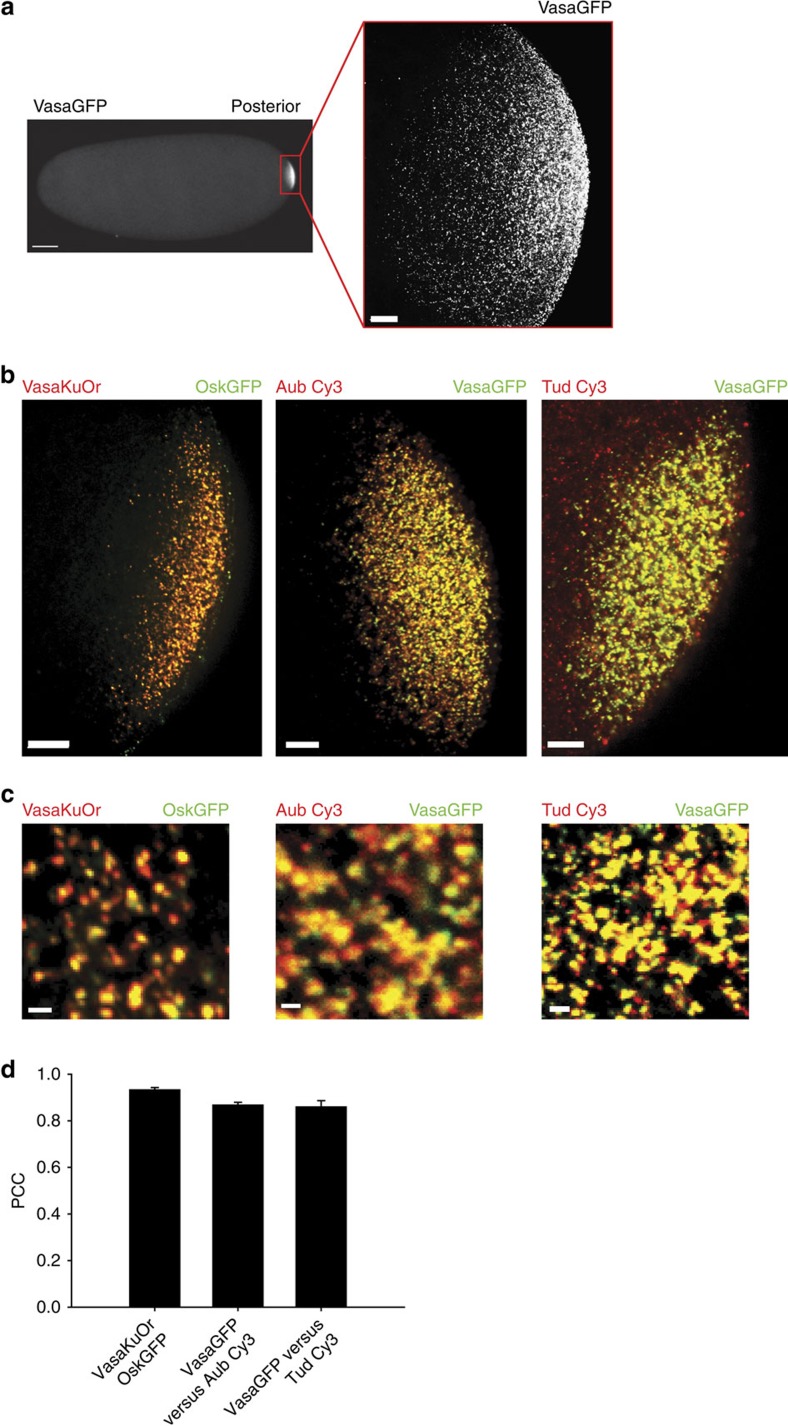
Germ plasm proteins occupy the same space within a granule. (**a**) A confocal image of an embryo expressing VasaGFP. (**b**,**c**) Images of embryos expressing VasaKuOr (red) and OskGFP (green) acquired with a widefield epifluorescence microscope, and a confocal image of an embryo expressing VasaGFP transgene (green) and immunostained against Aub (red) or Tud (red). In all panels embryos fixed at 0–1 h AEL were used. (**d**) PCC showing co-localization between Vasa, Osk, Aub and Tud. Nine, four and eight embryos were analysed for OskGFP/VasaKuOr pair, VasaGFP/Aub pair and VasaGFP/Tud pair, respectively. An average±s.e.m. is shown. Scale bar in (**a**) (embryo) 50 μm, in (**a**) (blow-up) and (**b**) 10 μm, in (**c**) 1 μm.

To determine the spatial relationship among protein components of germ granules, we chose Vasa, Oskar (Osk), Aubergine (Aub) and Tudor (Tud), known for their function in germ plasm assembly. We used protein fusions (Vasa green fluorescent protein (VasaGFP), Vasa Kusabira Orange (VasaKuOr), OskGFP) and immunostaining to visualize these proteins in the early embryo. The distribution of Vasa protein, detected by antibody staining, overlapped fully with that of VasaGFP and VasaKuOr transgenic fusion proteins, which allowed us to use these three probes interchangeably (Supplementary Fig. 1). In addition, Vas, Osk, Aub and Tud proteins were highly enriched at the posterior pole (Fig. 1a,b) and organized into larger, multi-protein particles (Fig. 1c). Given their size of up to 500 nm and round shape (Fig. 1c, Supplementary Fig. 2h–m, Supplementary Table 1) we concluded that the particles we detected by light microscopy corresponded to the electron-dense granules previously observed by electron microscopy (EM)^9^,^10^. Furthermore the Pearson correlation coefficient (PCC) analysis demonstrated that these proteins highly co-localized with each other (PCC>0.86; Fig. 1d), which was expected given that Vasa and Osk physically interact in the germ plasm granule^11^ while Aub physically interacts with both Vasa and Tud^12^. We conclude that germ plasm proteins occupy the same space and that this space most likely resembles germ plasm granules previously described by EM^1^.

### Germ granules are heterogeneous mRNA-protein aggregates

**Figure 2:**
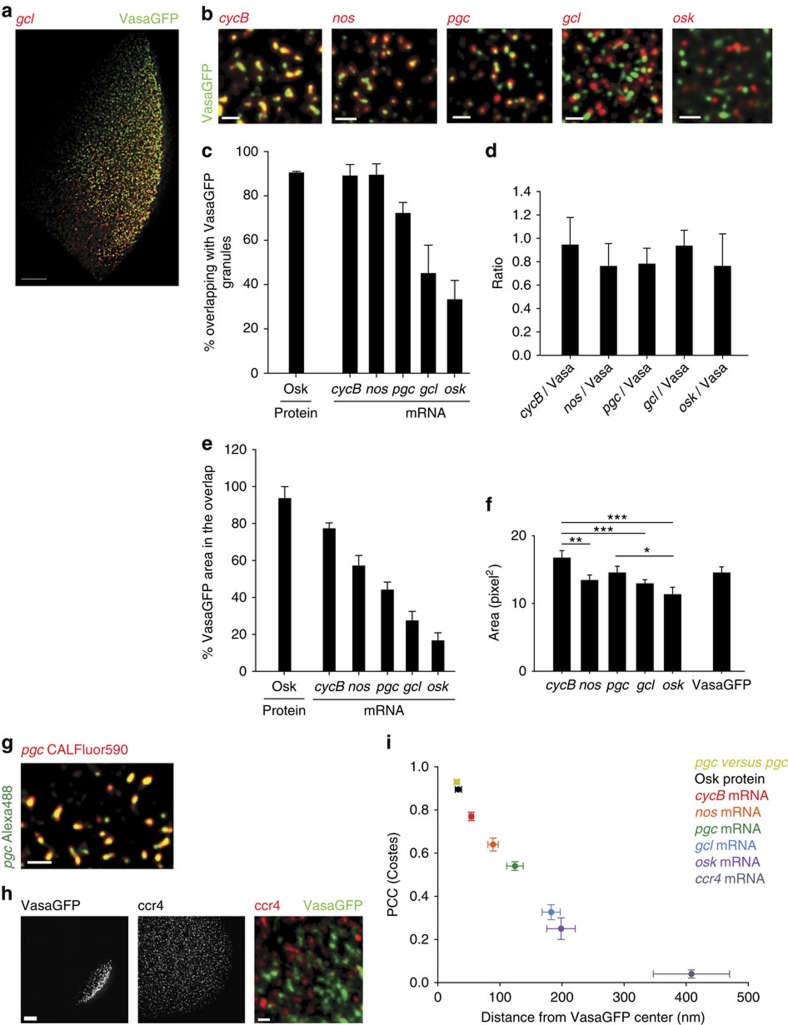
Germ plasm-enriched mRNAs occupy distinct positions within the VasaGFP granule. (**a**,**b**) SIM images of early *Drosophila* embryos (0–1 h AEL) expressing VasaGFP (green) and stained with CALFluor590-labelled smFISH probes, targeting respectively *cycB, nos*, *pgc, gcl* or *osk* (red). (**c**) Per cent of overlapping VasaGFP granules with mRNAs and VasaKuOr granules with OskGFP granules. An average±s.e.m. is shown. (**d**) Ratio between the number of *cycB*, *nos*, *pgc*, *gcl* and *osk* mRNA particles and VasaGFP granules obtained in c. For each embryo a ratio between the number of mRNA particles and VasaGFP was calculated. An average±s.d. is shown. (**e**) Per cent of VasaGFP or VasaKuOr area overlapping with *cycB*, *nos*, *pgc* or *gcl* mRNAs or OskGFP, respectively. Overlap measures the area of VasaGFP granule occupied by a corresponding overlapping mRNA particle. mRNA particles and granules in **c** were analysed. An average±s.e.m. is shown. (**f**) Size of localized mRNA particles and VasaGFP granules measured in pixels^2^ where pixel size X=Y=56 nm. An average±s.e.m. is plotted. *nos, gcl* and *osk* particles were smaller than *cycB* particles (**t-test, two-tailed *P*=0.01, ***t-test, two-tailed *P*=0.001). *osk* particles were smaller than *pgc* particles (*t-test, two-tailed *P*=0.03). (**g**) A SIM image of localized *pgc* mRNA labelled with a mix of Alexa488 (green) and CALFluor590 (red) smFISH probes. (**h**) A widefield epifluorescence image of *ccr4* mRNA (red) in embryo expressing VasaGFP (green). *ccr4* is not enriched at the posterior pole. (**i**) Co-localization of mRNAs within the VasaGFP granules, OskGFP granules within the VasaKuOr granules and Alexa488-labelled *pgc* mRNA within CalFluor590-labelled *pgc* mRNA determined by measuring the distance between the center of the VasaGFP granule and the center of the overlapping mRNA and by determining the PCC(Costes) (Methods section). An average±s.e.m. is plotted. Scale bar in **a**, **h** (right panel) 10 μm, in **b**, **g** and **h** (left panel) 1 μm.

Next we determined the distribution of the known germ plasm-enriched mRNAs *cycB*, *nos*, *pgc*, *gcl* and *oskar (osk)* and one control mRNA *ccr4*, which appears evenly distributed throughout the embryo^4^ (Fig. 2a,b,h). We employed single-molecule FISH (smFISH) (Fig. 2a,b, Supplementary Figs 2a–d,f and 5c) coupled with structured illumination microscopy (SIM)^13^, a super-resolution technique, to resolve the position of individual mRNAs in VasaGFP granules with sub-pixel resolution^14^ and determine the shape of mRNA particles within the granule (Methods section). We applied 100 nm TetraSpeck microspheres as alignment markers to correct pixel shifts, which aligned the protein and mRNA channels to a 14.8±1.4 nm precision (Supplementary Fig. 2e, Methods section). Thus, the combination of smFISH RNA detection with high-resolution imaging allowed us to determine the position of mRNAs within granules.

Using this method we asked how often mRNA particles overlapped with VasaGFP and how much of the granule area was contained by the mRNA particles. We established boundaries of mRNA and protein particles by image segmentation (Supplementary Fig. 2h–m, Supplementary Table 1) and determined the per cent overlap between VasaGFP and mRNA particles as well as between OskGFP and VasaKuOr (Fig. 2b,c). Consistent with the co-localization of germ plasm proteins (Fig. 1d) 90.3% of OskGFP overlapped with VasaKuOr (Fig. 2c). Similarly, 88.9% of *cycB* and 89.2% of *nos* particles overlapped with VasaGFP indicating that like OskGFP, *cycB* and *nos* populated the same granules. However, only 72.1, 44.9 and 33.1% of *pgc*, *gcl* and *osk* mRNA particles, respectively, overlapped with VasaGFP. This heterogeneity was not due to differences in the number of mRNA particles localized at the posterior pole since the ratio of VasaGFP particles to *cycB*, *nos*, *pgc*, *gcl* and *osk* particles was similar (Fig. 2d). Rather our results demonstrated that *cycB* and *nos* mRNAs exhibited a greater preference for co-localization with VasaGFP than *pgc*, *gcl* and *osk*. Germ granules are thus heterogeneous mRNA-protein aggregates.

In support, the per cent of the area of an individual VasaGFP granule that overlapped with an mRNA particle also varied, with *cycB* occupying 77.1% and *osk* mRNA occupying only 16.5% of the VasaGFP area (Fig. 2e, Methods section). *nos*, *pgc* and *gcl* mRNA particles were similar in size, while *cycB* mRNA particles were bigger and *osk* mRNA particles were smaller (Fig. 2f, Supplementary Table 1). Thus particle size could not account for the differences in overlap (Fig. 2e). Rather, our experiments suggest that *cycB*, *nos*, *pgc*, *gcl* and *osk* localize to different positions with respect to VasaGFP.

### mRNAs are spatially organized within granules

To directly assess the spatial organization of mRNAs in granules, we employed two measures of co-localization. First, we measured the distance in nm between the center of VasaGFP and the center of the overlapping mRNA (Supplementary Fig. 3a). Because VasaGFP and mRNAs were bright and almost perfectly circular (circularity>0.89, Supplementary Fig. 2m) we employed a spot detection algorithm to determine the position of an individual mRNA and VasaGFP and calculated the distance between them^14^ (see Methods section). Second, we limited the PCC analysis to overlapping mRNAs and VasaGFP using the Costes PCC approach (PCC(Costes))^15^ (Supplementary Fig. 3b–h). In contrast to the localization-based approach, PCC(Costes) is insensitive to the object shape; it ranges from 1 (perfect co-localization) to 0 (random-co-localization). It examines the spatial relationship between the intensities of two fluorescent objects rather than the frequency or duration of their co-occurrence^16^. Thus, objects that overlap frequently but not at a fixed distance will give a PCC(Costes) of 0 (Supplementary Fig. 3h). Furthermore, we used image randomization to statistically evaluate the likelihood of obtaining the measured PCC(Costes) by chance (Supplementary Fig. 3d,e, Methods section)^15^,^16^.

To determine the limits of co-localization detection we performed two control experiments. First, we labelled *pgc* with a mix of Alexa488-labelled and CALFluor590-labelled probes targeting overlapping regions on the mRNA (Fig. 2g). The distance between the center of Alexa488-labelled *pgc* and the center of CALFluor590-labelled *pgc* was 33.6±4.3 nm and the PCC(Costes) was 0.900±0.004 (Fig. 2i) and therefore represented the upper limit of co-localization detection. The deviation of co-localization between expected and observed was due to incomplete image alignment (Supplementary Fig. 2e) and image noise. The latter reduced the localization precision of mRNA clusters by 17.6±1.4 nm (Supplementary Fig. 4a,c). Second, we measured co-localization between the non-localizing *ccr4* mRNA and VasaGFP (Fig. 2h). *ccr4* was located far from the center of VasaGFP, co-localized with the granule by chance (distance 408.5±31.4 nm, PCC(Costes) 0.04±0.02; Fig. 2i) and therefore represented the lower limit of co-localization detection. Applying PCC(Costes) analysis to the germ plasm-enriched mRNAs, *cycB* was the most central within the VasaGFP granule (distance 53.9±3.4 nm, PCC(Costes) 0.77±0.02), while *osk* was the most peripheral (distance 198.4±22.9 nm, PCC(Costes) 0.25±0.05; Fig. 2b,i). These distances were significantly greater than the errors with which the position of each mRNA was determined (Supplementary Fig. 4b,c). In addition, the low variation in the distance measurements and a high PCC(Costes) suggest that the association of *gcl* and *osk* with VasaGFP did not occur by chance, as it was the case with *ccr4* mRNA (Supplementary Fig. 3b,e, Methods section). Rather, these two transcripts clearly localized at the edge of VasaGFP granules.

**Figure 3:**
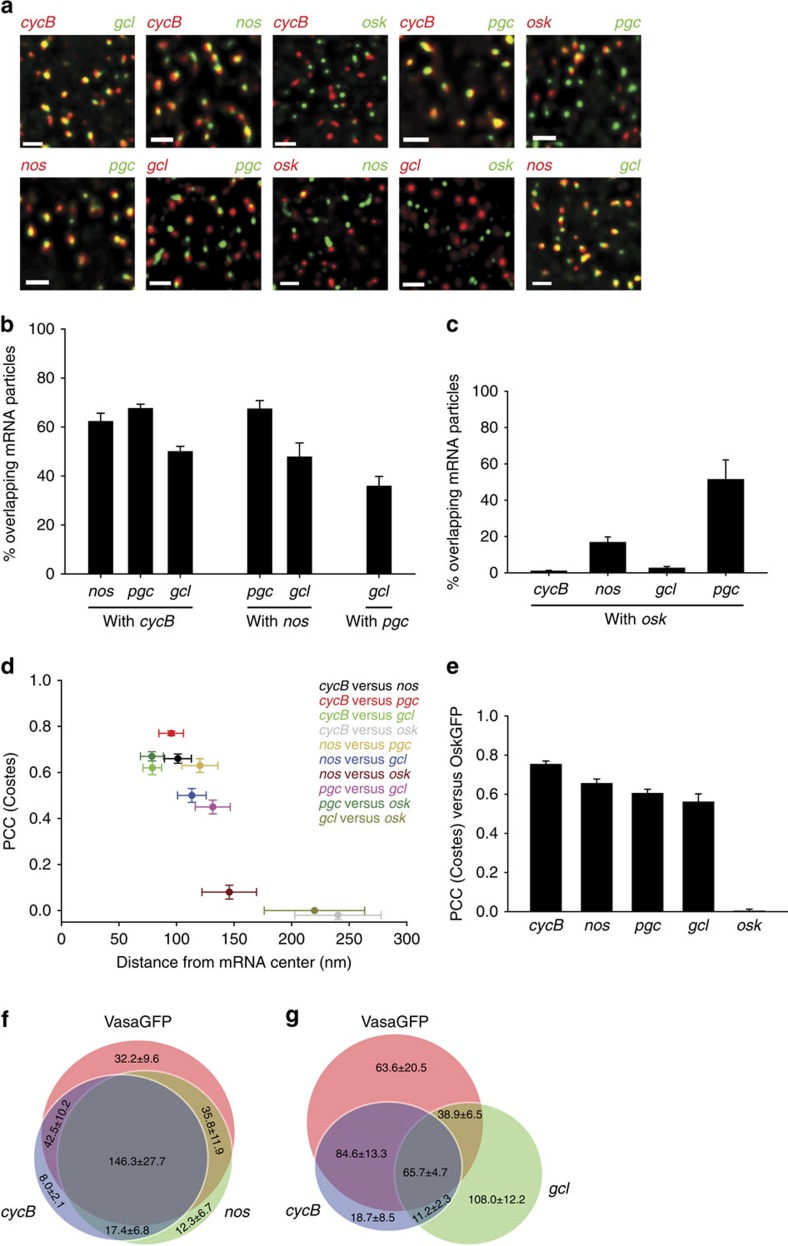
*gcl* but not *osk* is a constituent of the germ granule. (**a**) Two colour smFISH and SIM were used for detection of co-localized transcripts. (**b**) Per cent of overlapping mRNA particles as described in Fig. 2c. An average±s.e.m. is shown. (**c**) Per cent of overlapping *osk* mRNA particles with *cycB*, *nos*, *gcl* and *pgc* mRNA particles. An average±s.e.m. is shown. (**d**) Pairwise mRNA co-localization. Distance measurements and PCC(Costes) were performed as described in Fig. 2i. An average±s.e.m. is plotted. (**e**) Co-localization of *cycB*, *nos*, *pgc*, *gcl* and *osk* mRNA with OskGFP granules as quantified by PCC(Costes). An average±s.e.m. is plotted. (**f**,**g**) Venn diagrams showing concurrent overlapping of VasaGFP with *cycB* and *nos* and VasaGFP with *cycB* and *gcl*. Six and seven embryos expressing VasaGFP and double labeled to detect *cycB* and *nos* or *cycB* and *gcl* were analysed, respectively (Supplementary Fig. 3j). The numbers within Venn circles indicate number of VasaGFP granules and *cycB*, *nos* and *gcl* mRNA particles. An average±s.e.m. is shown. Scale bar in **a** 1 μm.

Our measurements strongly suggested that mRNAs are not randomly distributed within germ granules but that their distribution is structured. This structure could be due to mRNAs occupying different locations within the same VasaGFP granule or due to sorting of mRNAs to different VasaGFP granules that could overlap when in vicinity. In the latter scenario the mRNAs would overlap frequently, yet poorly co-localize as the overlap would be by chance. To distinguish between these possibilities, we asked how mRNAs were organized with respect to each other. By pairwise mRNA analysis we found that over 60% of *cycB* overlapped with *nos* and *pgc* while over 50% of *gcl* overlapped with *cycB* and *nos* (Fig. 3a,b). In addition, 56.9% of VasaGFP simultaneously overlapped with *cycB* and *nos* (Fig. 3f, Supplementary Fig. 4d) while 25.9% of VasaGFP concurrently overlapped with *cycB* and *gcl* (Fig. 3g, Supplementary Fig. 4d). *cycB*, *nos*, *pgc* and *gcl* also highly co-localized with each other indicating that their spatial relationship is not dictated by chance (Fig. 3a,d, Supplementary Fig. 3c,d, Supplementary Table 2). Thus, *cycB*, *nos*, *pgc* and *gcl* mRNAs populate the same germ granule, where *cycB* is positioned most central and *gcl* is located more peripheral.

Contrary, *osk* mRNA poorly overlapped with *cycB*, *nos* and *gcl* (Fig. 3c) with a PCC(Costes) close to 0 (Fig. 3d) indicating that *osk* co-localized with *cycB*, *nos* and *gcl* by chance. *osk* also randomly co-localized with OskGFP while *cycB*, *nos*, *pgc and gcl* highly co-localized with Oskar protein (Fig. 3e). Thus, *osk* mRNA is not a component of the germ granule supporting previous EM analysis^17^.

However, 33.1% of *osk* mRNA clusters also co-localized with VasaGFP (Fig. 2b,c) devoid of Osk protein, *cycB*, *nos*, and *gcl* mRNA (Fig. 3a–e). This suggests that *osk* mRNA can co-organize individually with certain germ granule components but not collectively within a germ granule, as defined by the presence of multiple core germ plasm proteins (Vasa, Tudor, Oskar, Aub; Fig. 1). Interestingly, *pgc* co-localized with *cycB*, *nos*, *gcl* and VasaGFP (Figs 2i and 3a,b,d, Supplementary Table 2) but also co-localized with *osk* with a PCC(Costes) of 0.67±0.02 (Fig. 3c,d, Supplementary Table 2). Thus *pgc* occupies two types of granules, one populated by core germ plasm proteins (Vasa, Tudor, Oskar, Aub) and mRNAs (*cycB, nos, gcl*) and the other populated by *osk* mRNA. Together, these results demonstrate that mRNAs localized to the posterior pole occupy distinct positions within granules and can also sort to different types of granules. Such a precise spatial organization was unanticipated by previous analysis.

### mRNAs are highly concentrated in germ plasm

**Figure 4:**
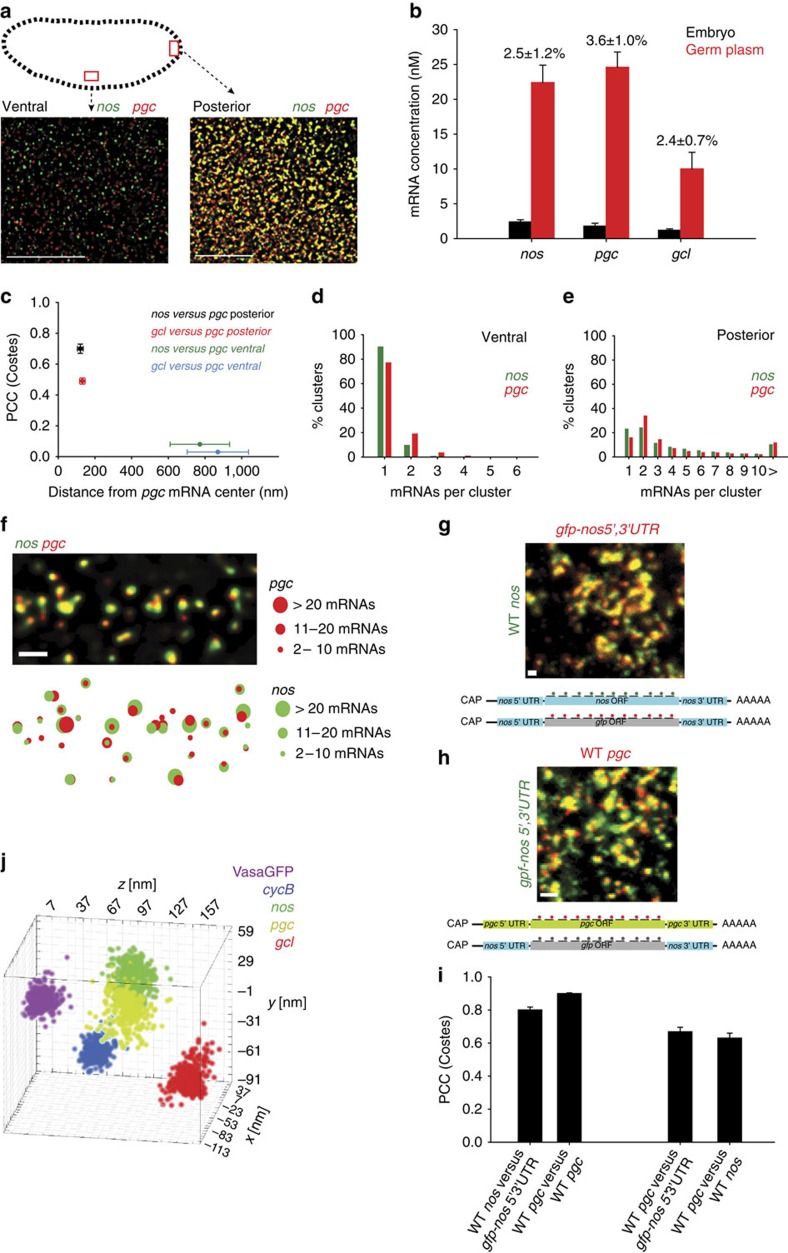
Localized mRNAs form homotypic rather than mixed clusters. (**a**) SIM and smFISH were used to detect *nos* (green) and *pgc* (red) mRNAs located ventrally and at the posterior of the embryo. (**b**) The concentration (nM) of *nos*, *pgc* and *gcl* mRNA found in the embryo (black bars) and localized at the posterior pole (red bars) determined by smFISH (Methods section). The localization efficiency of mRNAs at the posterior pole is indicated above the bars (Supplementary Fig. 5c,f–h, Supplementary Table 3 Methods section). Eleven embryos/mRNA were analysed. An average±s.e.m. is shown. (**c**) Co-localization of *nos* mRNAs and *gcl* mRNAs with *pgc* mRNAs ventrally and at the posterior of an embryo. An average±s.e.m. is shown. (**d**,**e**) Multiple *nos* or *pgc* mRNAs occupy a single *nos* or *pgc* mRNA cluster at the posterior. Ventrally *nos* and *pgc* were mostly found as single mRNAs. (**f**) A SIM image of *nos* (green) and *pgc* (red) mRNA with an accompanying spatial map of co-localization of homotypic *nos* (green) and *pgc* (red) mRNA clusters. The sub-pixel position of *nos* and *pgc* clusters and corresponding number of *nos* and *pgc* mRNAs per cluster was determined with Airlocalize (Methods section). (**g**) A confocal image of endogenous (wild type (WT)) *nos* mRNA (green) and the chimeric mRNA with GFP ORF and *nos* 5′ and 3′ UTRs (red) co-localizing at the posterior pole. Below the image is a depiction of both mRNAs labelled with either red or green smFISH probes. (**h**) A confocal image of WT *pgc* mRNA (red) and the chimeric mRNA with GFP ORF and *nos* 5′ and 3′ UTRs (green) co-localizing at the posterior pole. (**i**) Co-localization of WT *nos* mRNA with the chimeric GFP-*nos* 5′, 3′ UTR mRNA (four embryos), of WT *pgc* labelled with Alexa488 probes and WT *pgc* labelled with CALFluor590 probes (two embryos), of WT *pgc* and the chimeric GFP-*nos* 5′, 3′ UTR mRNA (six embryos) and WT *pgc* mRNA and WT *nos* mRNA (six embryos) quantified by PCC(Costes). (**j**) Using triangulation a 3D model of an average VasaGFP (pink) granule with localized *cycB* (blue), *nos* (green), *pgc* (yellow) and *gcl* (red) is shown (Methods section). Scale bar in **a** 5 μm, in **f**–**h** 1 μm.

To understand the organization of mRNA particles within the germ plasm better, we asked how efficiently these mRNAs localized at the posterior pole. We used smFISH to compare the concentration of *nos, pgc* and *gcl* at the posterior pole with that of the rest of the embryo. Outside of the posterior pole, *nos*, *pgc* and *gcl* are mostly found as single mRNAs (Fig. 4d, Supplementary Fig. 6b), consistent with the literature^18^. Despite different overall concentration, *nos*, *pgc* and *gcl* mRNAs localized with similar efficiencies, ranging from 2.4–3.6% (Fig. 4b, Supplementary Fig. 5, Supplementary Table 3, Methods section). Our measurements corresponded well with the 4% localization efficiency previously reported for *nos*^6^,^18^. The concentration of mRNAs in the germ plasm was also more than eightfold higher than elsewhere in the embryo. Thus, localized mRNAs are tightly packed into a small germ plasm volume.

To determine how such a dramatic change in mRNA concentration may affect the organization of individual mRNA molecules we analysed the spatial relationship between germ plasm-localized mRNAs and their ‘unlocalized' counterparts in the embryo (Fig. 4a). Outside of the posterior germ plasm area, *nos*, *pgc* and *gcl* were mostly found as single mRNAs (Fig. 4d, Supplementary Fig. 6b), overlapped infrequently and poorly co-localized (Fig. 4c, Supplementary Fig. 6a). These co-localization efficiencies were similar to those determined for *ccr4* mRNA in relation to VasaGFP (Fig. 2h,i). Thus, outside of the germ plasm *nos* and *gcl* overlapped with *pgc* by chance. At the posterior, however, the majority of *nos*, *pgc* and *gcl* were found in clusters containing more than one mRNA (Fig. 4e,f, Supplementary Fig. 6c). These mRNA clusters also more frequently overlapped and better co-localized (Fig. 3b,d) yet much less efficiently than *pgc*-Alexa488 co-localized with *pgc*-CALFluor590; on average, the distance between overlapping mRNA clusters was three times greater than observed for double-labelled *pgc* mRNA (Fig. 2g,i). Thus, once localized to the granules, *pgc* mRNAs preferred to co-organize with other *pgc* mRNAs rather than mix with *nos* or *gcl* mRNAs. Similarly, germ granule localized *nos* (or *gcl)* mRNAs preferred to co-organize with other *nos* (or *gcl*) mRNAs rather than mix with *pgc* mRNAs. These results suggest that germ granule-localized mRNAs organize into homotypic clusters.

### mRNAs form homotypic clusters within germ granules

To directly assess whether mRNAs group with each other into homotypic clusters, we measured co-localization between the endogenous *nos* and a chimeric *nos* construct, where the Nos protein coding sequence was replaced by GFP-Moesin^19^. The 3′ untranslated region (UTR) determinants that enrich *nos* mRNA at the posterior are present in both mRNAs and are regulated similarly^8^. Thus, if mRNAs organized into homotypic clusters, the mRNAs should co-habit the same space in the granule. The endogenous mRNA could be distinguished from the chimeric mRNA by smFISH probes detecting the Nos- and GFP-protein coding sequences, respectively (Fig. 4g). GFP signal did not obstruct the mRNA co-localization analysis (Supplementary Fig. 6d). We detected a high co-localization between the endogenous and the chimeric *nos* (PCC(Costes) 0.80±0.02; Fig. 4g,i), similar to the PCC(Costes) of *pgc*-Alexa488 co-localized with *pgc*-CALFluor590 (Fig. 2g,i). Consistent with the idea that each RNA cluster occupies its own space within an ribonucleoprotein granules granule, the relationship between *pgc* and the chimeric *nos* mRNA was similar to that of *pgc* and endogenous *nos* (PCC(Costes) 0.63±0.03 and 0.67±0.02, respectively; Fig. 4h,i). Therefore germ plasm-enriched mRNAs self-recognize and organize into homotypic clusters. To gain insight into how these homotypic clusters organized within a granule, we performed a triangulation analysis. Using the distance relationships established among VasaGFP and mRNA clusters (Figs 2i and 3d; Methods section) we were able to reconstruct the average structure formed by these clusters, confirming that they are confined to distinct three-dimensional (3D) volumes within a granule (Fig. 4j, Supplementary Fig. 6e,f, Supplementary Table 4). We conclude that despite being localized within the same granule, *nos*, *pgc* and *gcl* preferably grouped with mRNAs of their own kind to form homotypic clusters rather than mixed in heterotypic clusters.

### Position of mRNAs does not specify their translational onset

**Figure 5:**
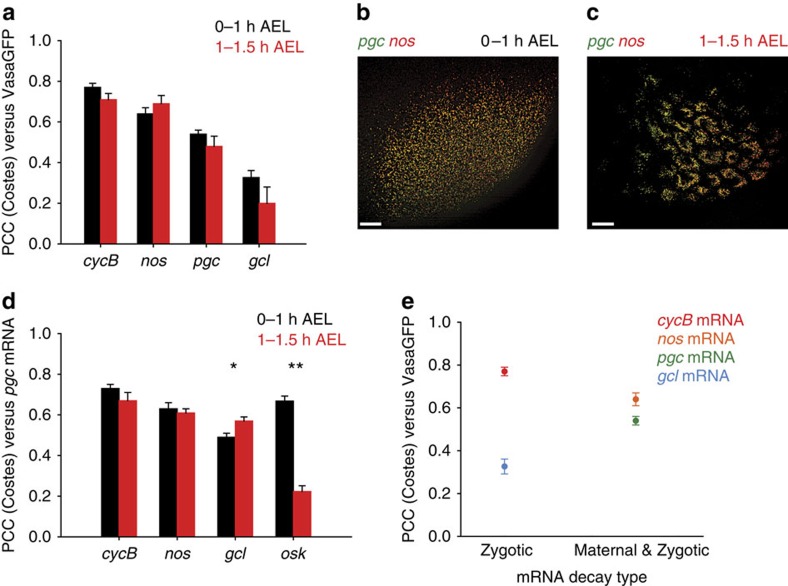
The position of mRNAs within the granule is independent of the translational or degradation onsets. (**a**) Embryos expressing VasaGFP transgene were fixed after 0–1 h AEL (black bars) or 1–1.5 h AEL (red bars) and smFISH for *cycB, nos*, *pgc* and *gcl* was performed. Black bars are PCC(Costes) measurements from Fig. 2i. For the PCC(Costes) analysis performed on 1–1.5 h AEL embryos (red bars), eight, nine, six and five embryos were analysed for the *cycB* mRNA/VasaGFP, *nos* mRNA/VasaGFP, *pgc* mRNA/VasaGFP and *gcl* mRNA/VasaGFP pairs, respectively. An average±s.e.m. is plotted. (**b**) SIM images of 0–1 h AEL *w*^*1118*^ embryo localizing *pgc* (green) and *nos* (red) mRNAs at the posterior pole. (**c**) SIM images of 1–1.5 h AEL *w*^*1118*^ embryo of *pgc* (green) and *nos* (red) mRNAs surrounding PGC nuclei. (**d**) *w*^*1118*^ embryos were fixed 0–1 h AEL (black bars) or 1–1.5 h AEL (red bars). A two colour smFISH was performed to detect *pgc* mRNA and either *cycB*, *gcl* or *osk* mRNA. Alexa488-labelled RNA probes were used to detect *pgc* mRNA and CALFluor590-labelled probes were used to detect *cycB*, *gcl* or *osk* mRNA (**b**,**c**). PCC(Costes) analysis was performed as described above. For the PCC(Costes) analysis performed on 0–1 h AEL embryos, 7, 7, 13 and 7 embryos were analysed for the *cycB* mRNA/pgc mRNA, *nos* mRNA/pgc mRNA, *gcl* mRNA/pgc mRNA and *osk* mRNA/pgc mRNA pairs, respectively. For the PCC(Costes) analysis performed on 1–1.5 h embryos, 7, 8, 10 and 7 embryos were analysed for the *cycB* mRNA/pgc mRNA, *nos* mRNA/pgc mRNA, *gcl* mRNA/pgc mRNA and *osk* mRNA/pgc mRNA pairs, respectively. An average±s.e.m. is plotted. **t-test, two-tailed *P*<0.0001, *t-test, two-tailed *P*=0.01. (**e**) In 0–1 h AEL embryos unlocalized *cycB* and *gcl* mRNAs are stable until the activation of zygotic genome∼2.5 h AEL (Zygotic) while unlocalized *nos* and *pgc* are unstable and decay before and during the activation of zygotic genome (Maternal & Zygotic)^24^. PCC(Costes) values were obtained in Fig. 2i. Scale bar in **b**,**c** 10 μm.

A likely hypothesis for the observed mRNA organization is that it may be related to mRNA regulation during germ cell development. Thus, we asked whether mRNA-protein structure could be linked to the translational onset of enriched mRNAs. Previous studies found that unlocalized mRNA was translationally repressed while localized mRNA became translated at the posterior pole^8^,^20^, with different timing of translational onset among localized transcripts^8^. We reasoned that the organization of transcripts within the granule could determine their translational activity. To test this hypothesis we looked at early embryos (0–1 h after egg laying (AEL)) where only *nos* mRNA was translationally active^8^,^20^. We found *nos* mRNA located in the center of the VasaGFP granule just like *cycB*, a translationally repressed mRNA^8^,^21^ (Fig. 5a, black bars) while translationally repressed *pgc* and *gcl* mRNAs^8^,^22^ were located at the periphery of the VasaGFP. No statistically significant change in the position of mRNA clusters within the granule was detected in older embryos (1–1.5 h AEL; Fig. 5a, red bars) in which *gcl* became translationally active^8^. In addition, the spatial relationship among different mRNA clusters also remained largely unaffected by the changes in translational activity (Fig. 5b–d, black and red bars, respectively). Only *gcl* mRNA shifted closer towards *pgc* mRNA, while *osk* mRNA moved away from *pgc*. We conclude that the position of mRNAs within the granule does not predict their translational onset.

Since the germ plasm protects localized mRNAs from degradation we reasoned, that the more susceptible an mRNA would be to decay in the embryo, the deeper in the granule it would be located. Degradation of maternally deposited *pgc,* and *nos* in the embryo is regulated by a ‘maternal' programme, which is active at the beginning of embryonic development and a ‘zygotic' programme, which is activated by zygotic transcription^23^,^24^, while *cycB* and *gcl* are degraded by the ‘zygotic' programme. Thus, *pgc* and *nos* may require more protection and be closer to the center of the granule than *cycB* and *gcl.* However, the position of *cycB*, *nos*, *pgc* and *gcl* mRNAs within the VasaGFP did not correlate with the ability of the germ plasm to protect these mRNAs from decay (Fig. 5e).

## Discussion

Here we combined single-molecule FISH with SIM, a super-resolution technique, to gain a high-resolution view of the mRNA-bound germ granule. This combinatorial approach allowed us to determine that germ granule-localized mRNAs occupy distinct positions within the granule and relative to each other, while germ granule proteins are homogeneously distributed within the granular space. Multiple localized mRNAs group to form homotypic cluster, which gives the germ granule its structure. This structure does not change through early embryonic development and does not correlate with the translational onset of localized mRNAs or with the ability of germ granules to protect bound mRNAs from decay.

We focused our analysis of the organizational structure of germ plasm on core germ granule protein components, Vas, Osk, Tud and Aub, and on *cycB*, *nos*, *pgc*, *gcl* and *osk* mRNA. *cycB*, *nos*, *pgc*, *gcl* and *osk* serve as prototypes for mRNA localization to the germ granules because their localization to the germ plasm, their regulation in the germ plasm and biological significance for germ cell biology are understood best. While mRNA localization studies suggest that up to 200 mRNAs may be localized to the posterior pole of the early embryo^2^,^4^, it is assumed that regulatory mechanisms revealed by the study of *cycB*, *nos*, *pgc* and *gcl* are shared among other germ plasm-localized mRNAs. The study of germ plasm-localized mRNA regulation revealed that only localized mRNAs translate while their unlocalized counterparts are translationally silent^8^,^20^,^26^,^27^,^28^,^29^,^30^, that localized mRNAs are protected from mRNA decay^31^ and that the 3′ UTRs of localized mRNAs are necessary and often sufficient to localize mRNAs to the posterior and render them translationally competent^8^. Our experiments demonstrate that *cycB*, *nos*, *pgc* and *gcl* mRNAs concentrate in homotypic clusters, assume specific positions within the germ granules, and can organize into separate granules. Our results make it unlikely that *cycB*, *nos*, *pgc* and *gcl* clusters contain more than one type of mRNA. If clustering between heterotypic mRNAs was a common organizational strategy, our pairwise analysis with *cycB*, *nos*, *pgc* and *gcl* would have not yielded the distinct volumes observed (Fig. 4j, Supplementary Movies 1 and 2). Thus, despite the fact that we only sampled a limited number of localized RNAs, we anticipate that germ granule organization observed for *cycB*, *nos*, *pgc* and *gcl* is also shared by other germ granule-localized mRNAs, which are similarly regulated.

Given that the core germ plasm proteins Osk, Vasa, Aub and Tud recruit other germ granule components^32^ and are themselves homogeneously distributed within the granule, it is unlikely that the germ granule structure is dictated by proteins alone. Homotypic clustering could also be driven by intramolecular RNA–RNA interactions, similar to those found in the localized *bicoid* mRNA at the anterior pole^33^ and in the co-packaged *osk* mRNA during transport to the oocyte posterior^34^. The dramatic increase in mRNA concentration in the granule compared with rest of the embryo (Fig. 4b, Supplementary Fig. 5i) may raise the likelihood for two mRNAs to interact or even induce RNA–RNA interactions by altering mRNA conformation thus driving homotypic clustering.

In yeast, the movement of mRNAs in and out of stress granules and processing bodies determines their translatability and stability and in *Drosophila* oocytes the position of *bicoid* and *gurken* mRNA within the sponge body correlates with their translational activity^25^,^35^. We find, however, that the mRNA position within the germ granule is independent of translational or degradation activity of localized mRNAs. Some translational and decay regulators found in germ granules are also found in sponge bodies, stress granules and processing bodies^2^. Thus our data imply that in germ granules these proteins may regulate transcripts differently to allow for the dynamic regulation of different mRNAs. Alternatively, sorting of mRNAs into distinct granules could specify their activity. For example, *pgc* co-localizes with core germ-granule components as well as with *osk* mRNA. Thus, the pool of *pgc* associated with *osk* could be functionally different from the one that associates with Vasa, Osk, *cycB*, *nos* and *gcl*. Indeed, in older embryos just before *pgc* becomes translated, *pgc* moves away from *osk*, but not from VasaGFP, *cycB*, *nos* and *gcl*. Speculatively, this could be the mechanism that determines the onset of *pgc* translation.

mRNA clustering could also enhance biochemical reactions locally either by enabling protein complex formation, by quick re-binding of a regulator to a neighbouring mRNA or by increasing the concentration of a regulator the cluster RNA codes for. For example, it has been proposed that the repression of *cycB* translation by Nanos protein (Nos) depends on a high local concentration of Nos in the germ plasm^21^. Multiple *nos* mRNAs within the cluster could increase the local concentration of Nos thus counteracting the loss of the unbound Nos due to diffusion into the embryo. Once bound to *cycB*, Nos could also be quickly re-bound by the neighbouring *cycB* mRNAs thus maintaining high Nos concentration and ensuring efficient *cycB* repression. In this way each mRNA cluster in the granule would resemble a biochemical territory, consistent with the recent observations showing that germ granules in *Caenorhabditis elegans,* which behave like liquid droplets, are also not homogeneous^36^,^37^. We propose that an mRNA-protein granule organization similar to the one described here for *Drosophila* germ granules could be a conserved feature of larger ribonucleoprotein granules.

## Methods

### Fly lines

The following *Drosophila* lines with the following maternal genotypes were used in this study: *w*^*1118*^ (‘wild type'; Bloomington Stock Center), *Δgcl*^38^, flies expressing a GFP-tagged Vasa transgene (y,w; P[E GFP- vas w^+^]^cyIII^; Lehmann lab, flies expressing a Kusabira Orange-tagged Vasa transgene (*UAS–vasa–ko*)^39^; flies expressing a GFP-tagged Oskar (pFlyFos-Osk^40^, gift from Pavel Tomancak), flies expressing a chimeric mRNA composed of the GFP ORF with *nos* 5′ and 3′ UTRs mRNA whose expression was driven by a *nos* promoter (nos-moe GFP (X))^41^.

### Single-molecule FISH

The smFISH protocol used in this study was a modification of protocols by Lécuyer *et al.* and Lionnet *et al.*^14^,^42^. Commercially available Stellaris RNA FISH probes labelled with CALFluor590, Quasar570 or Quasar670 were used for smFISH (Supplementary Tables 5–13). A mix of 48 3′ labelled probes hybridizing along the transcript strongly amplified signal-to-noise ratio and therefore our detection sensitivity. Alexa488 probes were labelled in the lab. These probes were obtained from IDT Technologies as 5′ end AmMC12 modified 20 nucleotide long DNA oligos and subsequently labelled with Alexa488 using the AlexaFluor 488 oligonucleotide Amine labeling kit (A-20191, Life Technologies). Uncoupled dye was removed with the MicroSpin G-25 Columns (27-5325-01, GE Heathcare). smFISH was then carried out as follows. Embryos collected 0–1 h AEL (or 1–1.5 h AEL for experiments in Fig. 5) were dechorionated and fixed for 20 min at room temperature (RT) in a scintillation vial filled with 5 ml of 4% paraformaldehyde and 1X PBS and 5 ml heptane saturated with 20% paraformaldehyde. Paraformaldehyde was removed and 5 ml of 100% methanol added. Vials were shaken vigorously for 15 s and embryos collected with a cutoff pipette tip. Embryos were washed three times with 100% methanol and stored overnight in 100% methanol at 4 °C. The next day ∼50 μl of embryos were rehydrated, once in a 1:1 mixture of methanol:PBT for 5 min and two times in PBT (5 min each; PBT solution: 1X PBS, 0.1% Tween-20). Embryos were then postfixed for 20 min in 4% paraformaldehyde and 1X PBS at RT, followed by three washes in PBT, each time for 2 min. Afterwards, embryos were treated with 3 μg ml^−1^ Proteinase K diluted in PBT, first at RT for 13 min and later for 1 h on ice. During incubations, embryos were mixed gently several times by inverting the tube. Proteinase K was removed and embryos washed twice with 2 mg ml^−1^ glycine. Embryos were postfixed again for 20 min in 4% paraformaldehyde and 1X PBS at RT and washed five times in PBT (2 min each). During a pre-hybridization step, embryos were incubated in 10% deionized formamide and 2 × SSC for 10 min at RT. Pre-hybridization solution was then removed, a hybridization mix containing smFISH probes added to embryos and incubated overnight at 37 °C. Per ∼50 embryos, we added 60 μl of hybridization mix composed of 10% deionized formamide, 1 μl of competitor (5 mg ml^−1^ *E. coli* tRNA+5 mg ml^−1^ salmon sperm ssDNA), 80 ng FISH probe mix, 10% of dextran sulphate, 2 mg ml^−1^ BSA, 2X SSC, 10 mM VRC and dH_2_O to 60 μl. Embryos and the hybridization mix were gently mixed by flicking the tube and incubated overnight in dark at 37 °C.

The next day, the hybridization mix was removed and embryos washed twice with 10% deionized formamide in 2X SSC pre-warmed to 37 °C for 15 min followed by two 1 h washes in 1X PBS. Embryos were mounted in ProLong Gold Antifade Reagent (P36934, Molecular Probes) containing a 10-fold dilution of 100 nm TetraSpeck microspheres (T-7279, Invitrogen).

### Microscopy and image processing

For SIM experiments, we used an instant SIM system, as previously described^13^. We used a 60X NA1.45 oil objective (Olympus), acquiring images in green or red channels with 488 and 561 nm excitation. To acquire confocal images, the Zeiss LSM780, AxioOberver microscope equipped with and argon laser, HeNe 633 laser, a DPSS 561-10 laser, a Plan-Apo40X/1.4 Oil DIC and EC Plan-Neofluar 10X/0.30 objectives was used. For Widefield Epifluorescence microscopy, API DeltaVision personalDV system equipped with Photometrics CoolSNAP HQ2 CCD camera and Olympus PlanApo N 60x/1.42 oil and UPlanSApo 20x/0.75 objectives was used.

All images acquired on SIM and widefield microscope were acquired in 3D, deconvolved and pixel shift-corrected in 3D using Huygens. All confocal images were acquired in 3D and pixel shift-corrected in 3D using Huygens.

### PCC and PCC(Costes)

PCC and PCC(Costes) quantify the degree of co-localization between imaged objects stained with spectrally distinct fluorophores^15^,^16^. They examine the relationship between the intensities of two fluorescent objects rather than the frequency or duration of their co-occurrence and are insensitive to object shapes^16^. PCC and PCC(Costes) were determined using the JACoP Plugin in ImageJ^16^. A 3D region of interest (ROI) located in the center of the posterior pole was analysed. Co-localization PCC ranges from 1 to −1, where 1 denotes perfect co-localization, 0 denotes co-localization that occurs by chance and −1 denotes exclusion. PCC(Costes) ranges from 0 to 1, where 0 indicates random co-localization while the value of 1 indicates high co-localization. In the Costes approach a threshold for each image was automatically set such that it minimized the contribution of noise to the correlation coefficient and enabled identification of overlapping (yellow) pixels independent of the user and independently of prior image segmentation. PCC(Costes) is insensitive to object shape or the object number variability between images and measures co-localization only between overlapping fluorescent signals (Supplementary Fig. 3b–h). Furthermore, significance of co-localization during the PCC(Costes) analysis was statistically evaluated by image randomization^15^,^16^. An image of the green channel (VasaGFP) was randomized by shuffling pixel blocks within the green image. Later, the PCC(Costes) between the randomized green image and the original red image (mRNA) was calculated. This process was repeated 200 times for a single green image, each time creating a different randomized image of the green channel and calculating a different PCC(Costes). These randomization results were then plotted to obtain the probability density of the PCC(Costes), which demarcated the extent of random co-localization for an individual green and red image pair (Supplementary Fig. 3d,e, blue curve). PCC(Costes) obtained from the original green and red images (Supplementary Fig. 3d,e, red line) was then compared with the probability density of the PCC(Costes) to evaluate, if the PCC(Costes) obtained indicated random co-localization. Finally, statistical significance (*P* value) of obtaining the PCC(Costes) determined for original green and red images by chance was calculated. The *P* value, expressed as a per cent, was inversely correlated with the probability of obtaining the PCC(Costes) determined for original green and red images by chance. For example, *P* value of 100% indicates that the likelihood of obtaining a particular PPC(Costes) by chance was minimal and that it is highly likely that the detected co-localization is indeed a *bona fide* co-localization^15^,^16^ (Supplementary Fig. 3d,e). For the PCC(Costes) analysis in Fig. 2i, six embryos were analysed for *cycB*, *nos*, *pgc*, and *osk* mRNA/VasaGFP pairs, four for *gcl*/Vasa GFP pair and two embryos for *ccr4*/Vasa GFP pair and *pgc* Alexa488/*pgc* CALFluor590 pair. Nine embryos were analysed for OskGFP granule and VasaKuOr granule. In Fig. 3e, 9, 11, 11, 12 and 11 OskGFP-expressing embryos were analysed for *cycB*, *nos*, *pgc*, *gcl* and *osk* mRNA, respectively.

### Determining overlap and ratio between mRNAs and VasaGFP

Two-dimensional ROI were analysed. For each ROI a threshold was first determined to allow subsequent segmentation of mRNAs and granules in ImageJ (Supplementary Fig. 2h–l). Segmentation was performed based on the fluorescent intensity of mRNA particles and granules and not based on their shape. All mRNAs and granules were analysed, regardless of their shape. Segmented particles were analysed using Analyze Particles Plugin in ImageJ. The ratio between the mRNA particles and VasaGFP granules for each ROI was determined, after which an average ratio was calculated. In Fig. 2c six embryos were analysed for *cycB*, *nos*, *pgc* and *osk* mRNA/VasaGFP pairs, four for *gcl* mRNA/VasaGFP pair and nine for OskGFP/VasaKuOr pair. For each ROI between 62 and 1,189 mRNA particles/granules were analysed. Over 3,500 particles were analysed to determine the size of mRNAs and granules in Fig. 2e. In Fig. 3b, 7 embryos were analysed to determine the overlap of *cycB* with *nos, pgc* or *gcl* and *nos* with *gcl* or *pgc* and 13 to determine the overlap of *pgc* with *gcl*. Each ROI contained between 26 and 1,388 mRNA particles. In Fig. 3c, seven embryos were analysed to determine the overlap of *osk* with *pgc*, six to determine the overlap of *osk* with *nos* or *gcl* and five to determine the overlap of *osk* and *cycB*. Each ROI contained between 48 and 686 mRNA particles.

### Determining the VasaGFP granule area in the overlap

The per cent of overlap measures the area of an individual VasaGFP granule occupied by an overlapping mRNA particle. For each mRNA/VasaGFP pair, OskGFP/VasaKuOr pair and *pgc* Alexa488/*pgc* CALFluor590 pair, objects were first segmented as described above. The overlap between objects (Supplementary Fig. 3a) was then determined using Image Calculator in ImageJ and subsequently the VasaGFP area in the overlap determined using Analyze Particles Plugin.

### Single mRNA counting

To circumvent issues associated with imaging and analysing very large image data sets, we acquired representative 3D ROI of a known volume as proxies for the concentration of unlocalized mRNAs across the entire embryo. We selected regions either ventrally or at the posterior, just outside of the germ plasm (Supplementary Fig. 5a). Single mRNAs were then counted in the 3D image stack using a spot detection algorithm (Airlocalize)^14^ and the concentration of mRNAs was determined. Briefly, the spot detection algorithm uses a 3D Gaussian kernel to find the center and intensity of each spot (the kernel is calculated based on the average point spread function shape)^43^. The robustness of spot localization against high fluorescent background (that is, created by neighbouring particles in a crowded environment) is enhanced by an affine local background subtraction before applying the Gaussian kernel. To determine the absolute number of transcripts in an embryo we determined the volume of an embryo and then extrapolated the number of transcripts obtained from a 3D ROI onto the entire embryo (Supplementary Fig. 5b). To determine the absolute number of mRNAs per germ granule-localized mRNA cluster, we determined the cumulative fluorescent intensity of an individual mRNA cluster and normalized it by the fluorescent intensity value of a single mRNA located outside of the embryo posterior. A series of control experiments increased our confidence of single mRNA detection; high signal-to-noise increased detection sensitivity (Supplementary Figs 2a,b and 4a–c), smFISH detected mRNA specifically (Supplementary Fig. 2c,d,f), mRNA levels determined by smFISH were in a good agreement with relative mRNA expression levels determined by RNA-seq (Supplementary Fig. 5c).

### Distance measurements

The spot detection algorithm, Airlocalize, was used to fit the intensity distribution of each fluorescent spot to a Gaussian curve and measured the position of the spot center with sub-pixel resolution (Supplementary Fig. 3a). Two-dimensional ROIs were analysed. mRNAs and protein granules identified as overlapping with VasaGFP (Fig. 2c,g) or with other mRNAs (Fig. 3b) were analysed. About 48, 48, 51, 47 and 55 mRNA/VasaGFP pairs were analysed for *cycB, nos*, *pgc, gcl* and *osk,* respectively. Overlapping 35 OskGFP granule/VasaKuOr granule pairs were analysed, 20 for *ccr4* mRNA clusters/VasaGFP pairs and 22 for *pgc* mRNA/*pgc* mRNA pair. After the position of each mRNA or germ granule was determined, the shortest distance in nm between overlapping mRNAs and granules was calculated. In Fig. 3d, minimally 31 overlapping mRNAs were analysed. In Fig. 4c 48, 45, 26 and 30 *pgc* mRNAs overlapping with *nos* mRNA ventrally, *nos* mRNA at the posterior, *gcl* mRNA ventrally and *gcl* mRNA at the posterior were analysed, respectively.

### Determining the localization precision

The accuracy with which the center of mass can be measured is determined by the signal-to-noise ratio in an image. To address the contribution of signal-to-noise to the localization precision of *cycB*, *nos*, *pgc*, *gcl* and *osk* mRNAs relative to VasaGFP center we performed the following experiment. smFISH and CALFluor590 probes used in Fig. 2b,i were employed to individually detect localized *cycB*, *nos*, *pgc*, *gcl* and *osk* mRNAs in *w*^*1118*^ flies. For each mRNA, an embryo posterior was imaged in 3D (Z step=200 nm, 20 steps). The same stack was acquired sequentially 12 times. After deconvolution, a small ROI cropped from the middle plane was analyzed in all 12 imaged stacks and the position of mRNA particles determined using a spot detection algorithm (Supplementary Fig. 4a). Afterwards, 16 distances between the same neighboring mRNA particles were measured in all 12 images (for example in Image 1 in Supplementary Fig. 4a marked as (d_1_)_1_, (d_2_)_1_,… (d_16_)_1_ and in Image 2 marked as (d_1_)_2_, (d_2_)_2_,… (d_16_)_2_). Finally, the localization precision was determined by subtracting distances in Image 2–12 from the same distances in Image 1 (for example (d_1_)_2_—(d_1_)_1_, (d_1_)_3_—(d_1_)_1,_….,(d_1_)_16_—(d_1_)_1_)). The values were then averaged and plotted with a s.e.m. in Supplementary Fig. 4c. All mRNAs were imaged with the same acquisition parameters. To determine the localization precision in a two color image we used Alexa488-labeled *pgc* and CALFluor590-labeled *pgc* and *w*^*1118*^ embryos. Embryo posterior was imaged in 3D (Z step=200 nm, 20 steps) sequentially 10 times. All 3D stacks were deconvolved and afterwards pixel shift-corrected as described in Supplementary Fig. 2e. A small ROI cropped from the middle plane was analyzed in all 10 stacks and the position of red and green *pgc* particles determined (Supplementary Fig. 4b). Afterwards, 10 distances between the co-localizing *pgc* particles were measured in all 10 images (for example in Image 1 in Supplementary Fig. 4b marked as (d_R1-G1_)_1_,… (d_R10-G10_)_1_ and in Image 10 marked as (d_R1-G1_)_2_,… (d _R10-G10_)_10_). Finally, the localization precision was determined by subtracting distances in Image 2–10 from the same distances in Image 1 (for example (d_R1-G1_)_2_—(d_R1-G1_)_1_,…., (d _R10-G10_)_10_)—(d_R10-G10_)_1_). The values were then averaged and plotted with an s.e.m. in Supplementary Fig. 4c. Precision errors with which individual mRNA particles were localized are significantly smaller than the distances between the centers of *cycB*, *nos*, *pgc*, *gcl* or *osk* mRNA particles and the center of VasaGFP (Fig. 2i).

### Determining mRNA localization efficiency

The posterior pole of an embryo was imaged in 3D such that whole volume of VasaGFP was captured. The total amount of mRNA fluorescence located within the cumulated VasaGFP signal was calibrated by the intensity of a single mRNA located outside of the posterior pole (Supplementary Fig. 5a) to obtain the total number of mRNAs localized and by the VasaGFP volume to determine the concentration (nM) of localized mRNAs (Fig. 4b, Supplementary Fig. 5f, Supplementary Table 3). Thus a 3D image of the VasaGFP signal was used as a mask, which was then applied onto an mRNA channel to determine the amount of fluorescence located within the VasaGFP mask. The VasaGFP 3D mask was created with the 3D Object Counting Plugin in Fiji^16^. Because *pgc* and *gcl* localized at the periphery of the VasaGFP, the segmentation of their FISH signal by the VasaGFP signal was less efficient than that of *nos* mRNA. We measured that 91.8±4.6, 86.4±9.7, 87.6±7.9 and 72.2±2.8 per cent of *cycB*, *nos*, *pgc* and *gcl* mRNA signal was segmented by the VasaGFP signal (Supplementary Fig. 5g,h). The localization efficiency determined by the VasaGFP signal was therefore corrected for this segmentation deficiency.

### Triangulation of germ granule components

Using triangulation, we reconstructed the average structure of the germ granules based on pairwise measurements between homotypic clusters (Figs 2i and 3d, Supplementary Table 2). Starting from a randomly generated set of (*x*, *y*, *z*) spatial coordinates for each of the five considered particles (VasaGFP and *cycB*, *nos*, *pgc* or *gcl* mRNA), we calculated the coordinate set that best matched the measured set of 10 average pairwise distances using the quasi-Newton minimization method. We verified that the optimization results were insensitive to the starting set of random coordinates. To further confirm the validity of the result, we computed the average deviation of the pairwise distances calculated on the average set of coordinates from those measured (8.2 nm, Supplementary Table 4), a value within experimental uncertainty (16.8 nm, which corresponds well with the 17.6±1.4 nm localization precision determined in Supplementary Fig. 4b,c).

To take into account the effect of our experimental resolution, we then repeated the same minimization 1,000 times, each time using a set of pairwise distances drawn from a normal distribution centred on the measurement mean, with standard deviation equal to the measurement uncertainty. The set of coordinates obtained at each iteration by quasi-Newton minimization were robustly registered onto the original coordinates by finding the optimal combination of 3D translation and rotations using the Random Sample Consensus technique.

The resulting 1,000 structures were found to partition equally into two classes of spatial models (Supplementary Movies 1 and 2, model 1: *n*=500; model 2: *n*=500; model 2 plotted in Fig. 4j). Each structure was automatically assigned to each model class using k-means clustering. The two classes of models were similar, differing mainly in their chirality: the position of the *gcl* and VasaGFP particles around the plane formed by *cycB*, *nos* and *pgc* are inverted between model 1 and 2 (Supplementary Movies 1 and 2). The center of each cloud of points represents the average center of VasaGFP granule or *cycB*, *nos*, *pgc* and *gcl* mRNA cluster. The extent of each cloud of points reflects the uncertainty of the position of each cluster within a germ granule, based on the measurement resolution and do not reflect the 3D size of the VasaGFP granule or an mRNA particle.

We omitted *osk* from our calculations because *osk* mRNA cluster poorly overlapped and randomly co-localized with OskGFP protein as well as *cycB*, *nos* and *gcl* mRNA clusters (Fig. 3c–e), suggesting it was not a consistent component of the germ granules. Adding *osk* into the coordinates calculations did not affect the results (we obtained two classes of structures with opposing chiralities resembling those obtained without *osk*). However, the structures where *osk* was included gave a poorer fit to the data (22 nm average deviation from the measured distances with *osk*, against 8.2 nm without *osk,* (Supplementary Table 4)), reinforcing the idea that *osk* is not a stable component of the germ granules.

All computations were performed using a custom-written Matlab code (Mathworks), and spatial structures were visualized using ViSP^44^.

### Immunofluorescence

Embryos expressing VasaGFP were immunostained as previously described^45^ to detect Vasa and Tudor. A rabbit polyclonal antisera directed against the N-terminal 16 AA of Aub was raised and affinity purified as described by Brennecke *et al.*^46^. A 1:5,000 dilution of the primary antibody was used to detect Vasa, Tudor and Aubergine.

## Additional information

**How to cite this article:** Trcek, T. *et al.* *Drosophila* germ granules are structured and contain homotypic mRNA clusters. *Nat. Commun.* 6:7962 doi: 10.1038/ncomms8962 (2015).

## Supplementary Material

Supplementary Information Supplementary Figures 1-6, Supplementary Tables 1-13 and Supplementary References

Supplementary Movie 1 A 3D model of the VasaGFP granule with localized *cycB*, *nos*, *pgc* and *gcl* determined by triangulation of the VasaGFP:mRNA and mRNA:mRNA distances (Methods). Each point in the plot represents the center of the VasaGFP granule or *cycB*, *nos*, *pgc* and *gcl* mRNA cluster. The extent of each cloud of points reflects the most likely position of each VasaGFP center or the most likely position of the center of an mRNA cluster within a germ granule, based on the measurement resolution and do not reflect the 3D size of the VasaGFP granule or an mRNA particle.

Supplementary Movie 2 A 3D model of the VasaGFP granule with localized *cycB*, *nos*, *pgc* and *gcl* determined by triangulation of the VasaGFP:mRNA and mRNA:mRNA distances (Methods, Supplementary Movie 2). The model is similar to the one shown in the Supplementary Movie 1 but differs from it in its chirality.
