# Supplementary material for: Drosophila germ granules are structured and contain homotypic mRNA clusters
Source: Nat Commun. 2015 Aug 5;6:7962. doi: 10.1038/ncomms8962 (PMC4918342; doi:10.1038/ncomms8962)
Supplement: Supplementary Information — Supplementary Figures 1-6, Supplementary Tables 1-13 and Supplementary References [file ncomms8962-s1.pdf]

SUPPLEMENTARY FIGURES

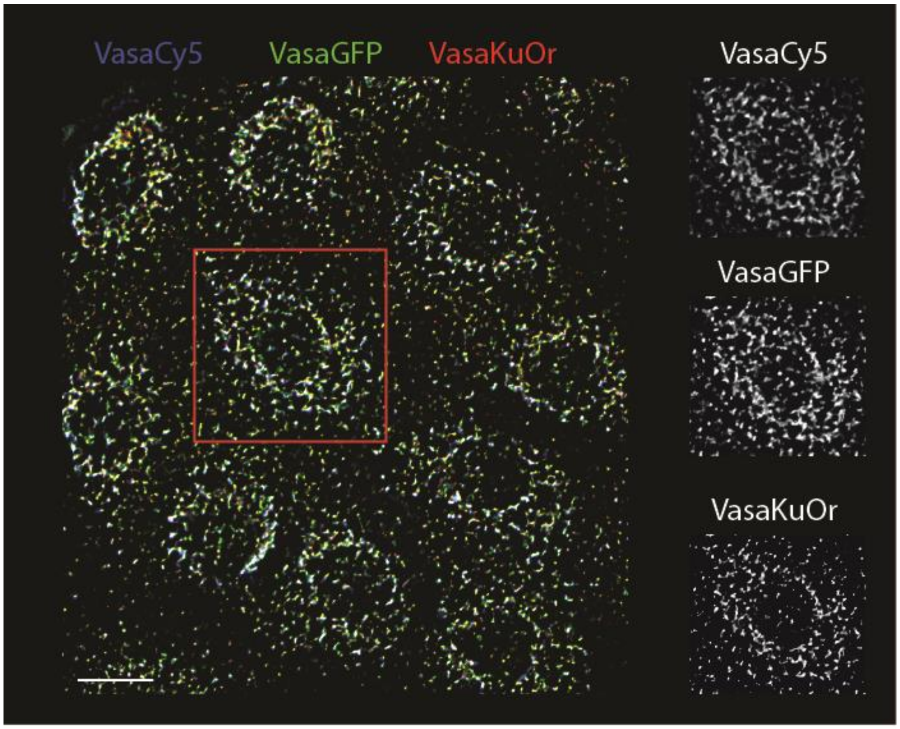

Supplementary Figure 1. Transgenic VasaGFP reflects the pattern of endogenous Vasa. A widefield epifluorescence image of an embryo expressing VasaGFP and VasaKuOr and immunostained to detect endogenous Vasa protein. Embryos were fixed 1 to 1.5h AEL. In these embryos the nuclei already migrated to the surface of an embryo. At the posterior pole the germ plasm is organized in crescents around the nuclei. Scale bar 10 µm.

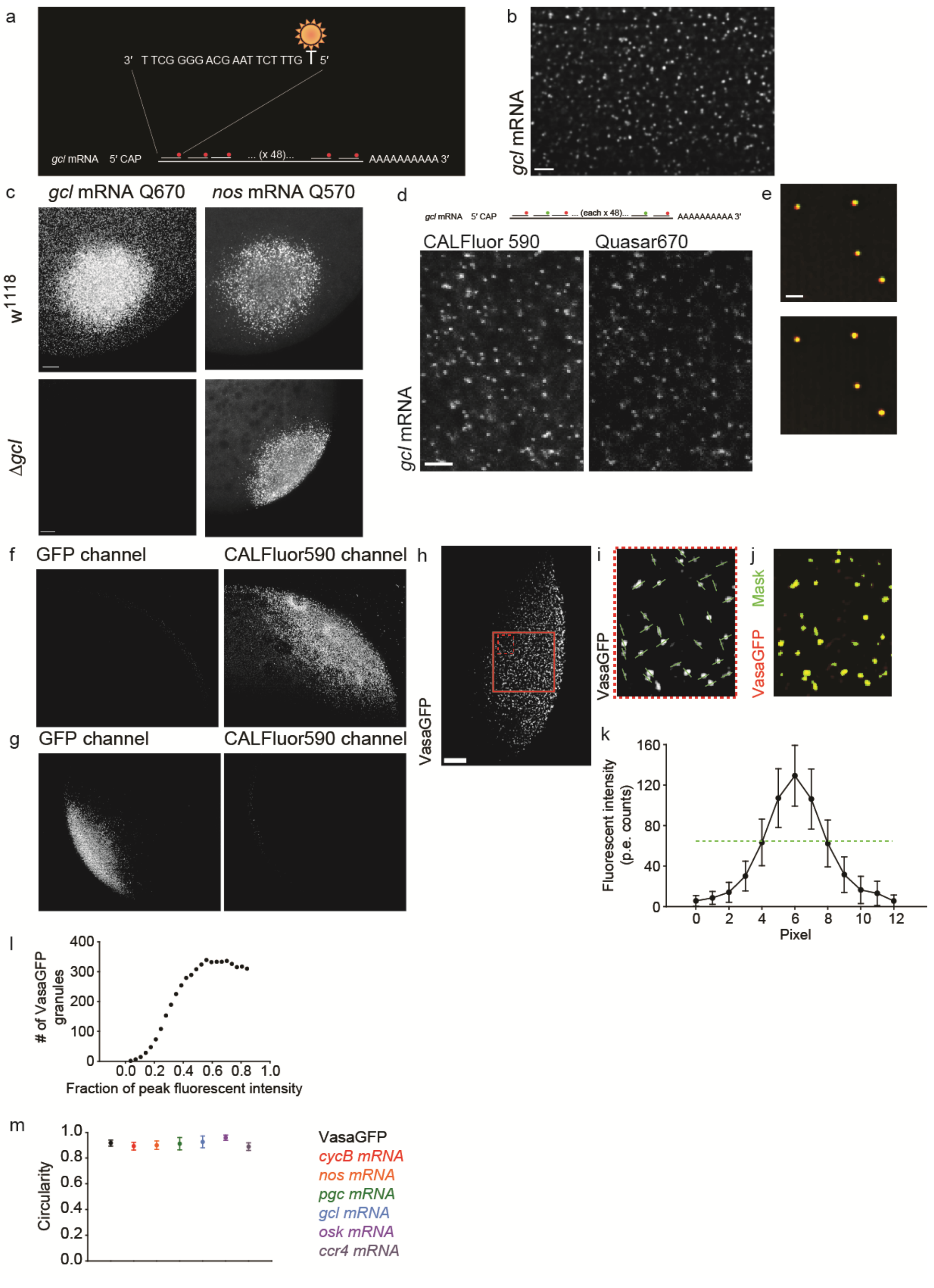

Supplementary Figure 2. smFISH enables analysis of mRNA co-localization with the germ granules. **(a)** A schematic of *gcl* mRNA labeled with smFISH probes. **(b)** A SIM image of *gcl* mRNAs found ventrally in the embryo. Scale bar 2 µm. **(c)** smFISH is specific and distinguishes between *nos* and *gcl* mRNAs in w^{1118} (WT) embryo. In the

absence of *gcl* expression in *Δgcl* embryo, only *nos* mRNA could be detected. Widefield images are shown. **(d)** A confocal image of a *gcl* mRNA labeled with spectrally distinct CALFluor590 and Quasar670 smFISH probes. The two fluorescent signals overlapped. Scale bar 2 µm. **(e)** Pixel shift-correction using 100 nm TetraSpeck microspheres before and after pixels-shift correction (Methods). Pixel shift correction aligned the images to 14.8±1.4 nm precision. Scale bar 1 µm. **(f,g)** SIM images demonstrating that there was no signal bleedthrough between channels during image acquisition. In f, w^{1118} embryos were used. In g, embryos expressing VasaGFP were used. **(h-l)**. Determining the segmentation threshold. For each embryo, a 2D ROI (solid red lines in h) was analyzed. Lines were drawn through at least 25 different mRNAs or granules and their fluorescent profiles determined (i and red square drawn with dashed lines in h) using ImageJ. All mRNA particles and granules were considered, regardless of the magnitude of their fluorescence intensity or their shape. (j) VasaGFP granules (red) overlaid with the segmentation mask of the VasaGFP (green). (k) An average profile of the fluorescent intensity ± s.e.m. of the VasaGFP granule. A green line demonstrates a half-maximal value of this profile, which was used for thresholding during segmentation. p.e. denotes photoelectron counts. (l) At half-maximum the number of detected mRNA particles or granules in an image no longer changed. **(m)** Circularity of VasaGFP granules and mRNA particles. Once the segmentation mask was created for each mRNA particle or VasaGFP the shape of the mRNA particles and granules was determined by analyzing the segmentation mask using Analyze Particles plugin in ImageJ. An average ± s.e.m. is shown. n > 3500 mRNA particles or VasaGFP granules. Scale bar in h 10 µm.

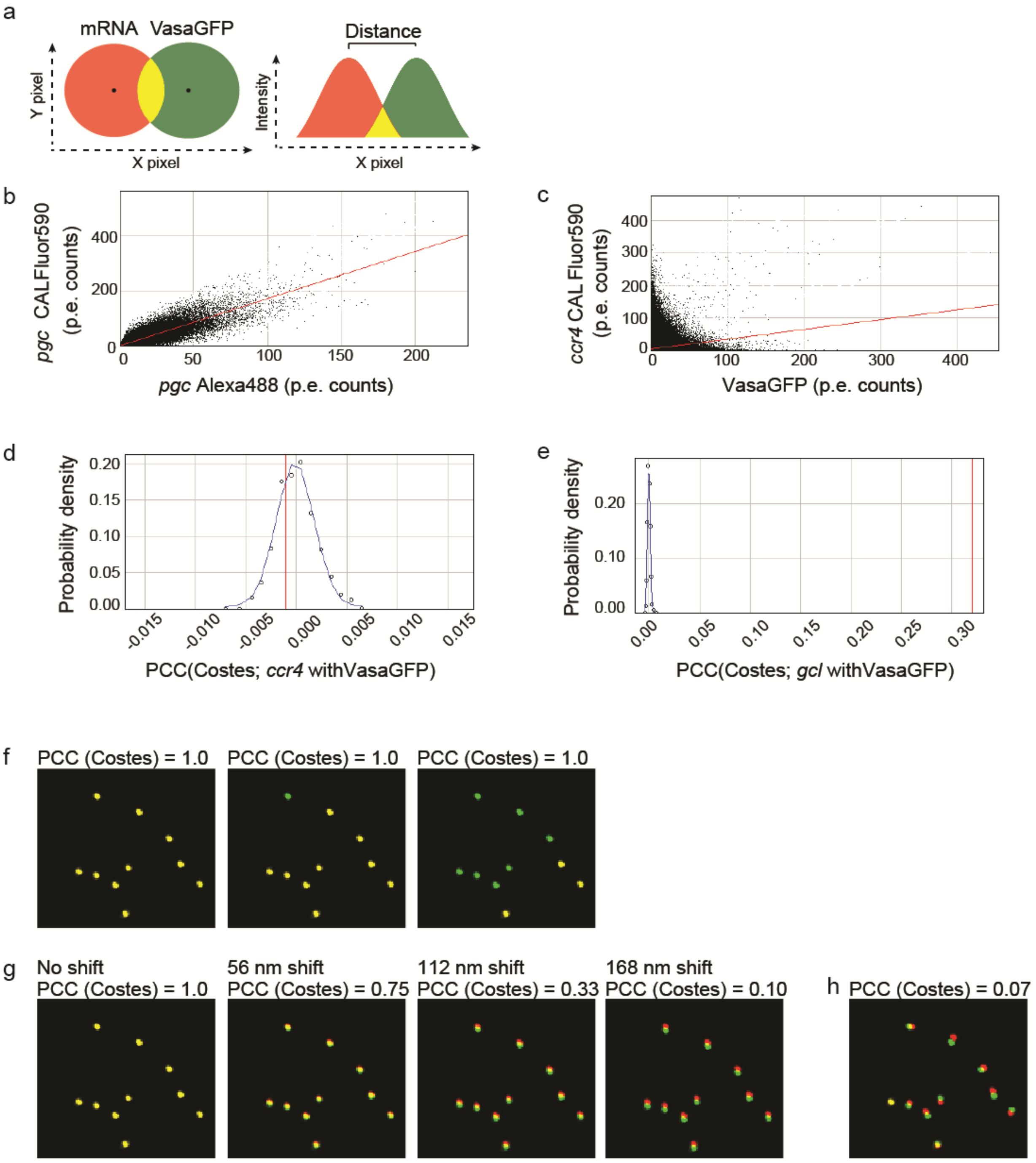

Supplementary Figure 3. Co-localization analysis using distance measurements and PCC(Costes) analysis. (a) A schematic depicting quantification of particle overlap (yellow), percent particle area in the overlap, distance measurements and PCC(Costes) measurements (Methods). **(b,c)** Cytofluorograms obtained during PCC(Costes) analysis of the *pgc* Alexa488 mRNA co-localizing with *pgc* CALFluor590 mRNA (PCC(Costes) 0.89) and *ccr4* mRNA co-localizing with VasaGFP (PCC(Costes) 0.088). In the case of high co-localization (b), high fluorescent signal in one channel correlates with the high fluorescent signal in the other channel, while low fluorescent signal in one channel also

correlates with the low fluorescent signal in the other channel. In the case of random co-localization (c), high fluorescent signal in one channel correlates both with low and high fluorescent signals in the other channel and *vice versa*. Red line in b,c demonstrates the fit to the data to obtain the correlation coefficient. **(d,e)** Co-localization of *ccr4* mRNA but not of *gcl* mRNA with VasaGFP occurs by chance. Plotted is the distribution of the PCC(Costes) of randomized images (blue curve) and the non-randomized image (red line) for *ccr4* mRNA (d) and *gcl* mRNA (e) co-localizing with VasaGFP (Methods). PCC(Costes) for *ccr4* was similar to that obtained during image randomization with a p-value of the PCC(Costes) of 30.9% indicating, that the likelihood that the co-localization of *ccr4* with the VasaGFP was non-random was very small. In contrast, PCC(Costes) for *gcl* was much higher than the one obtained during image randomization with a p-value of the PCC(Costes) of 100 % indicating a high likelihood of a bona fide co-localization between *gcl* and VasaGFP. **(f)** PCC(Costes) was insensitive to object number variability. **(g)** A shift of one pixel (56 nm) causes a change in the value of PCC(Costes) to 0.75, while a shift of 3 pixels (193.5 nm) decreases it to 0.10. PCC(Costes) (Methods). **(h)** A model illustrating random co-localization with a PCC(Costes) of 0.07. Objects in h overlap as frequently as those in g yet not at a fixed distance and will therefore give a PCC(Costes) of 0 (Methods).

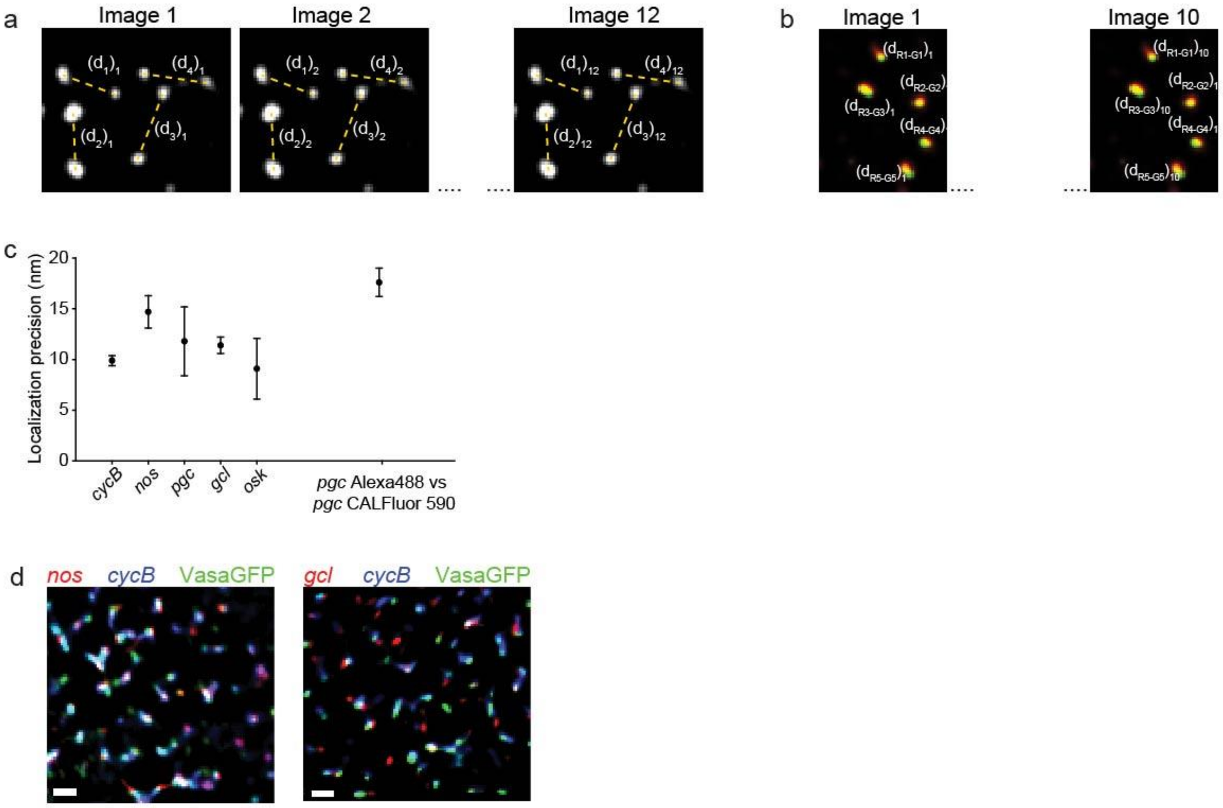

Supplementary Figure 4. Co-localization analysis reveals that germ granule-localized mRNAs occupy distinct positions within a granule. **(a)** Determining the localization precision (Methods). Images depict distances between the neighboring mRNA particles used for the localization precision error calculation (in Image 1 marked as $(d_1)_1$, $(d_2)_1$,… (d_{16})1 and in Image 2 marked as $(d_1)_2$, $(d_2)_2$,… $(d_{16})_2$). **(b)** Determining localization precision in a two color image (Methods). Images depict distances between the neighboring mRNA particles used for the localization precision error calculation (in Image 1 marked as $(d_{R1\text{-}G1})_1$,… $(d_{R10\text{-}G10})_1$ and in Image 10 marked as $(d_{R1\text{-}G1})_2$,… $(d_{R10\text{-}G10})_{10}$). **(c)** Localization precision of smFISH-labeled *cycB*, *nos*, *pgc*, *gcl* and *osk* mRNA using CALFluor590 probes and of smFISH-labeled pgc mRNA using Alexa488 and CALFluor590 probes. Precision errors with which individual mRNA particles were localized are significantly smaller than the distances between the centers of *cycB*, *nos*, *pgc*, *gcl* or *osk* mRNA particles and the center of VasaGFP (Fig. 2i). **(d)** A widefield image of a two color smFISH in embryo expressing VasaGFP. Quasar570 probes were used to detect *nos* or *gcl* mRNAs and Quasar670 probes were used to detect *cycB* mRNA. Scale bar 1 µm.

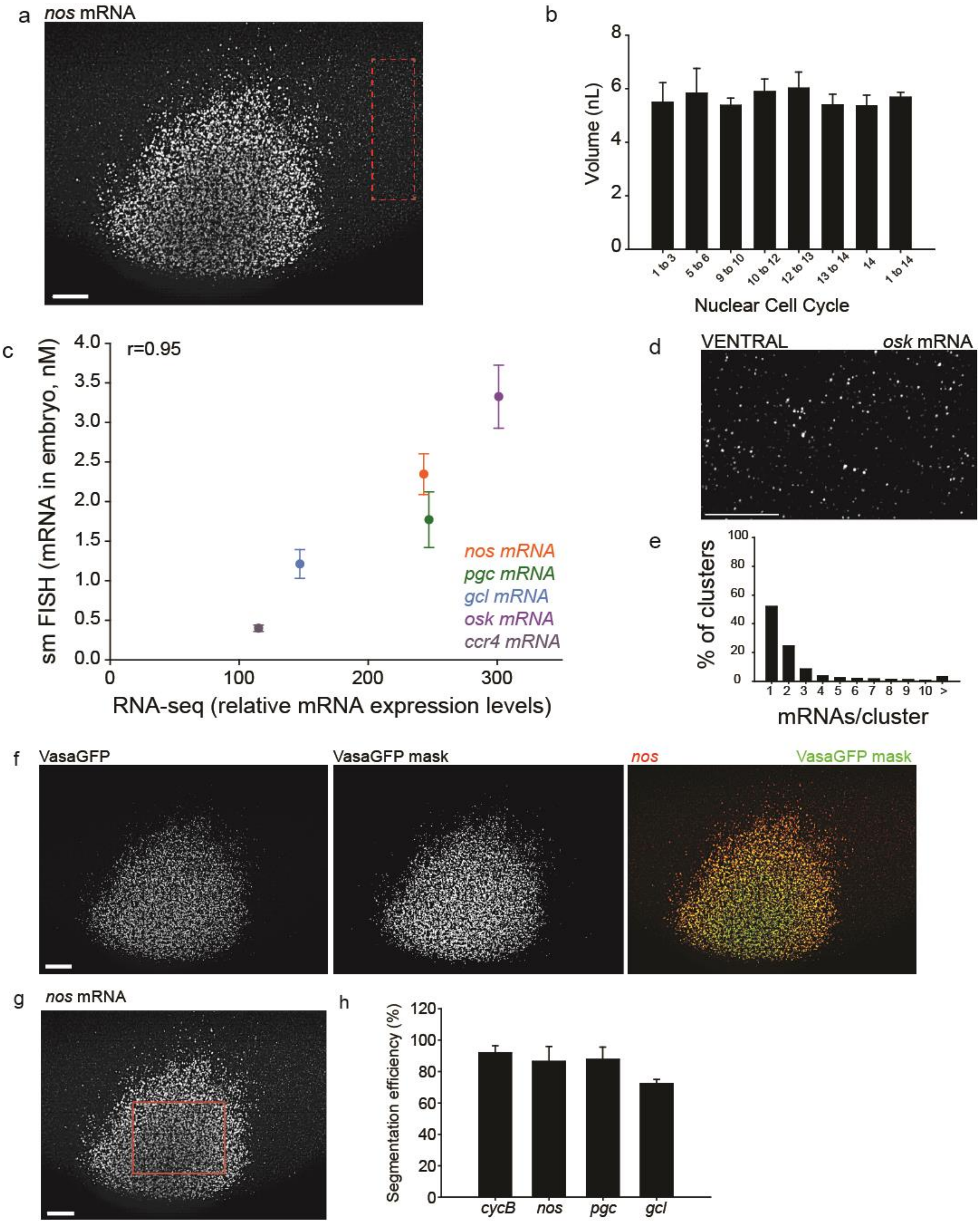

Supplementary Figure 5. Germ plasm-localized mRNAs are tightly packed into a small germ plasm volume. (a) To determine the number of mRNAs in an embryo by smFISH, single mRNAs were first counted in a 3D ROI of a known volume located

ventrally or at the posterior pole outside of the germ plasm (dashed red line). By calculating the volume of an embryo **(b)**, the number of mRNAs per embryo could then be determined. The volume of an embryo was determined as previously described[47]. An average volume of an embryo was 5.67±0.7 nL similar to published values[47] and did not change during early embryogenesis. Volume was determined in embryos during the first 14 nuclear cell cycles. 0 to 1h AEL time point contains embryos belonging to the nuclear cell cycle 1 to 8, while 1 to 1.5h AEL time point contains embryos belonging to the nuclear cell cycle 9 to 10. **(c)** Correlation between relative expression levels and nanomolar (nM) concentration of *ccr4*, *gcl*, *pgc*, *nos* and *osk* mRNA levels in the embryo determined by RNA-seq (FlyBase[48]) and smFISH, respectively. Correlation factor between the two datasets was r = 0.95. 10, 11, 11, 11 and 3 embryos were analyzed for *ccr4*, *gcl*, *pgc*, *nos* and *osk*, respectively. An average molarity (nM) ± s.e.m. is shown. **(d)** A SIM image of *osk* mRNA located ventrally in the embryo. **(e)** smFISH revealed that approximately 50 percent of unlocalized *osk* mRNAs were present in clusters containing more than one *osk* mRNA. **(f)** mRNA localization efficiency to the posterior pole was determined by using Vasa GFP as a mask during segmentation (Methods). **(g)** To determine how effective was the segmentation of the fluorescent signal of localized mRNAs by VasaGFP, we first measured the total fluorescence of the mRNA signal in a 2D ROI and compared it to the total fluorescence of the mRNA signal obtained when the mRNA signal in the same 2D ROI was segmented using the VasaGFP mask. The difference plotted in (h) was then used to correct localization efficiency quantified in f. Scale bar in a,f,g 10 µm and in d 2 µm.

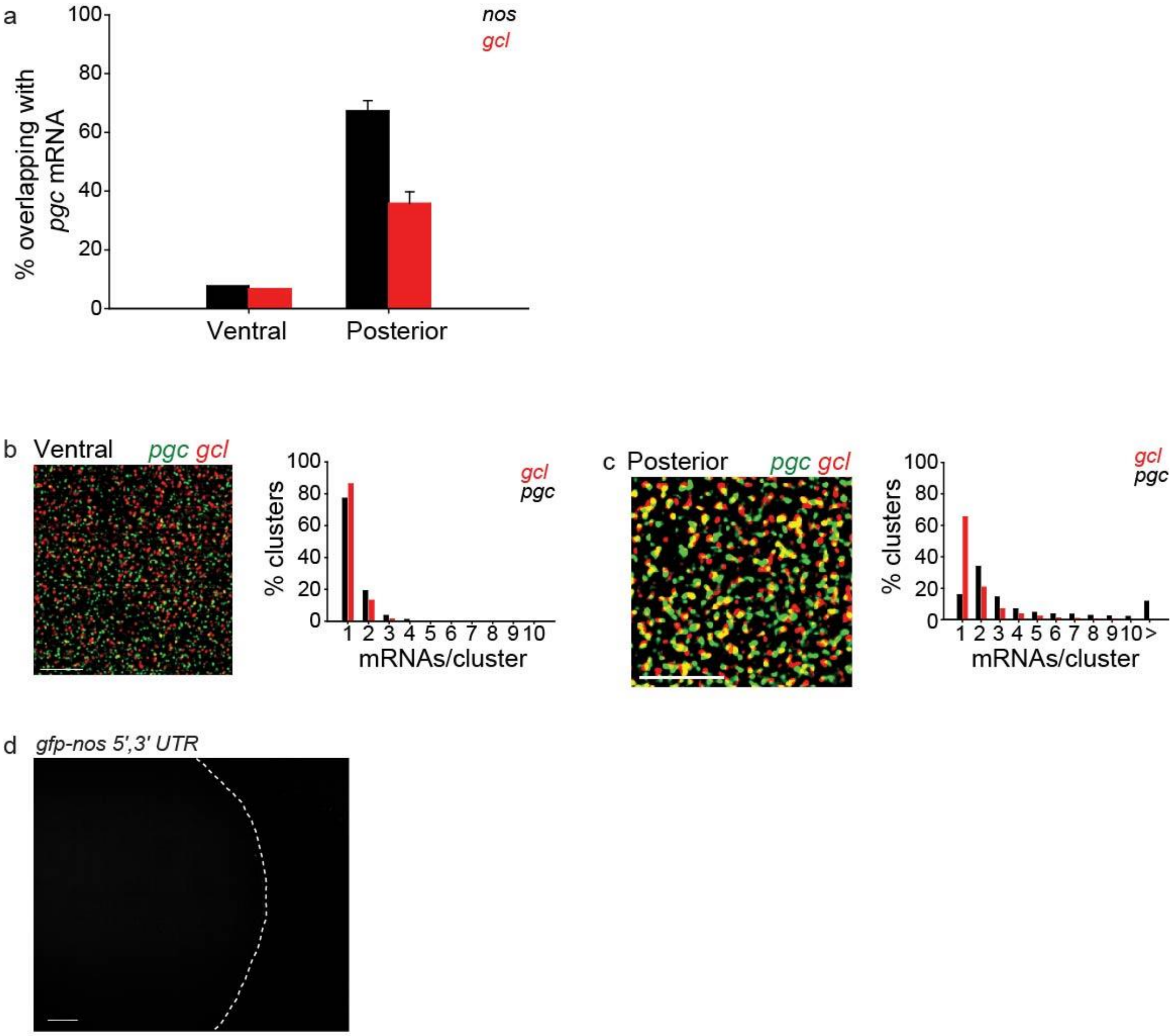

Supplementary Figure 6. Germ granule-localized mRNAs are organized into homotypic mRNA clusters. (a) The percent of overlapping *nos* mRNAs (black bars) and *gcl* mRNAs (red bars) with *pgc* mRNAs ventrally and at the posterior of an embryo. The overlap was determined as described in Fig. 2c. The measurements of overlaps found at the posterior are from Fig. 3b. To determine overlaps ventrally, between 178 and 1299 *nos*, *gcl* and *pgc* particles were analyzed. **(b,c)** Co-localization of *gcl* mRNAs (red) with *pgc* mRNAs (green) ventrally (b) and at the posterior (c) of an embryo. smFISH analysis demonstrated that at the posterior pole there were multiple *nos* or *pgc* mRNAs localized within *nos* or *pgc* mRNA clusters while ventrally they were mostly found as single mRNAs. **(d)** A SIM image of an embryo expressing the chimeric *nos* mRNA (*gfp-nos 5′, 3′ UTR*). Embryos were subjected to a smFISH procedure while withholding the smFISH probes. The GFP signal coded by the chimeric mRNA was too weak to be detected by SIM and thus did not interfere with pairwise mRNA co-localization analysis. Dotted line demarcates the edge of the embryo. Scale bar in b,c 2 µm and in d 10 µm.

SUPPLEMENTARY TABLES

Sizes of mRNA particles and VasaGFP granules

	cycB	*nos*	*pgc*	*gcl*	*osk*		VasaGFP
Average (nm^2)	52277.12	42187.73	45676.17	40376.2	35515.13		45603.28
% of cycB	100.0	80.7	87.4	77.2	67.9		87.2
% of nos		100.0	108.3	95.7	84.2		108.1
% of pgc			100.0	88.4	77.8		99.8
% of *gcl*				100.0	88.0		112.9
% of *osk*					100.0		128.4
% of VasaGFP	114.6	92.5	100.2	88.5	77.9		100.0

Supplementary Table 1

Pairwise mRNA co-localization parameters

	Distance (nm)	PCC(Costes)
cycB:nos	101.2±11.7	0.66±0.02
cycB:pgc	95.4±10.7	0.77±0.01
cycB:gcl	78.9±8.1	0.62±0.01
nos:pgc	120.3±15.6	0.63±0.03
nos:gcl	113.3±12.4	0.5±0.03
pgc:gcl	131.5±15.2	0.45±0.03
osk:cycB	240.2±37.5	-0.02±0.02
osk:nos	145.8±23.7	0.08±0.03
osk:pgc	78.6±10.0	0.67±0.02
osk:gcl	219.8±31.3	0±0

Supplementary Table 2

mRNAs localize with the same efficiency

	nos	*pgc*	*gcl*
# mRNA/embryo ($*10^6$)	8.0±0.9	6.1±1.2	4.1±0.6
mRNA conc./embryo (nM)	2.4±0.3	1.8±0.4	1.2±0.2
Localization efficiency	2.5±1.2	3.6±1.0	2.4±0.7
mRNA conc. at the posterior (nM)	22.4±2.5	24.6±2.2	10.0±2.4

Supplementary Table 3

Triangulation

	Data		Structure including *osk*			Structure excluding *osk*		
	**Measured Pairwise Distance (nm)**	**Measurement Uncertainty (nm)**	**Pairwise Distance on calculated Structure (nm)**	**Deviation Model / Measurement**	**z-score Model / Measurement**	**Pairwise Distance on calculated Structure (nm)**	**Deviation Model / Measurement**	**z-score Model / Measurement**
cycB* : *nos	101.2	11.7	86.6	14.6	1.25	92.0	9.2	0.79
cycB* : *pgc	95.4	10.7	110.4	15	1.40	96.6	1.2	0.11
cycB* : *gcl	78.9	8.1	101.1	22.2	2.74	95.8	16.9	2.09
nos* : *pgc	120.3	15.6	127.9	7.6	0.49	120.8	0.5	0.03
nos* : *gcl	113.3	12.4	129	15.7	1.27	119.6	6.3	0.51
pgc* : *gcl	131.5	15.2	110.9	20.6	1.36	130.6	0.9	0.06
osk* : *cycB	240.2	37.5	200	40.2	1.07	n/a	n/a	n/a
osk* : *nos	145.8	23.7	196.4	50.6	2.14	n/a	n/a	n/a
osk* : *pgc	78.6	10	95	16.4	1.64	n/a	n/a	n/a
osk* : *gcl	219.8	45	159	60.8	1.35	n/a	n/a	n/a
**VasaGFP: *cycB***	53.9	3.4	71.7	17.8	5.24	71.6	17.7	5.21
**VasaGFP: *nos***	88.9	8.3	93.2	4.3	0.52	96.0	7.1	0.86
**VasaGFP: *pgc***	124.2	13.3	131.7	7.5	0.56	123.0	1.2	0.09
**VasaGFP: *gcl***	182.5	14.6	166.4	16.1	1.10	161.6	20.9	1.43
**VasaGFP: *osk***	198.4	22.9	223	24.6	1.07	n/a	n/a	n/a
**Average**		**16.83**		**22.27**	**1.55**		**8.19**	**0.49**

Supplementary Table 4. An average germ granule was reconstructed twice, once with *osk* mRNA and once without *osk* mRNA. Including *osk* in the triangulation calculations gave similar results to those presented in Supplementary Movie 1 and 2; the structures cluster into two groups with opposing chiralities. However, the predicted distances were a much better match to the data when omitting *osk*; an average 8.2 nm deviation from the measured data versus an average 22 nm deviation from the measured data when *osk* mRNA cluster was included.

cycB smFISH probes

Probe Sequence (5' to 3')	Probe name
cgttttgttgttgcctccat	CycB CalFluor590_1
tatgccgcgattctgcaaat	CycB CalFluor590_2
tctttctgtgccgcatcctt	CycB CalFluor590_3
tctgtgagcttgagatcctt	CycB CalFluor590_4
tccacccgagctttggcatt	CycB CalFluor590_5
agtggctgtttcttccagtg	CycB CalFluor590_6
ttgccattgccattggtgct	CycB CalFluor590_7
aattcgaacgcaaaaacgcc	CycB CalFluor590_8
tacagtggtcttggtcggaa	CycB CalFluor590_9
tgactgtaactttagtgggt	CycB CalFluor590_10
ttcacgttctcggaagaact	CycB CalFluor590_11
ttgctgtcctcgcgctttaa	CycB CalFluor590_12
agtttggtcagcgacttctt	CycB CalFluor590_13
attcctgaaactcccatcac	CycB CalFluor590_14
aaacagctactggttcccgt	CycB CalFluor590_15
ttcttggtctctgcctcttt	CycB CalFluor590_16
cttcttggtttctggcagtt	CycB CalFluor590_17
cctttttcacttccagtgag	CycB CalFluor590_18
tgcactgttgcccctaataa	CycB CalFluor590_19
atcgtagatgtggtcgtagt	CycB CalFluor590_20
ttgctggaaagggacatggt	CycB CalFluor590_21
atcaatgtcctcgattccag	CycB CalFluor590_22
accaggttctccttgtcatt	CycB CalFluor590_23
cgtttacatattcggagacc	CycB CalFluor590_24
cctgatacaagtagtcgtag	CycB CalFluor590_25
atccttgtgaatgggctgct	CycB CalFluor590_26
atcttgtgggacacctcctt	CycB CalFluor590_27
ttgatccaatcgatcagcac	CycB CalFluor590_28
atggaactgcaggtggactt	CycB CalFluor590_29
tagcgatcaatgatagccac	CycB CalFluor590_30
tttggtgtccttgaccacct	CycB CalFluor590_31
actcccaccaattgcaagta	CycB CalFluor590_32
acttggtggctatgaagagt	CycB CalFluor590_33
aagacgaaatctccgattgc	CycB CalFluor590_34
agtgtaggtgtcgtccgtga	CycB CalFluor590_35
agatttgcagctccatctgt	CycB CalFluor590_36
agattacagtcgatggcctt	CycB CalFluor590_37
tagcgtcgaaggaagtgaat	CycB CalFluor590_38
ttggacatcgtatggtgctc	CycB CalFluor590_39
ggaagctaactcgatgaagt	CycB CalFluor590_40
taagtggccatttcgtagtc	CycB CalFluor590_41
aacagtgaggcagctgcaat	CycB CalFluor590_42
tttccattgagcaagtgcag	CycB CalFluor590_43
acggtcgttgaatcctgtac	CycB CalFluor590_44
atcgcgagtagaaggtcaga	CycB CalFluor590_45
ttcgcgatcagccgggtaat	CycB CalFluor590_46
ttgtagatggccttcagctt	CycB CalFluor590_47
atcttctggaacttgctgcc	CycB CalFluor590_48

Supplementary Table 5

nos smFISH probes

Probe Sequence (5' to 3')	Probe name
ggtaaagctacgcgccaact	Nos CalFluor590_1
acggcaattccaggaatttt	Nos CalFluor590_2
acttgaaacaactgcgaagc	Nos CalFluor590_3
aaaatcgtatgtcccttaga	Nos CalFluor590_4
aaatcgtgacgcagaggcaa	Nos CalFluor590_5
aactaaactcgcttttgggt	Nos CalFluor590_6
ttcgcgatacttctttatct	Nos CalFluor590_7
gcacagtttattcaactgaa	Nos CalFluor590_8
caaatcctcacccaaaaacc	Nos CalFluor590_9
ttatcgcgcactctactttc	Nos CalFluor590_10
aaatccgggtcgaaagttac	Nos CalFluor590_11
taaacgctgcaaaagctgcc	Nos CalFluor590_12
gctttgatcggaatgcgtat	Nos CalFluor590_13
ccaggcgctatttaaacgtt	Nos CalFluor590_14
gcacgggataacgctctaaa	Nos CalFluor590_15
agtgatcgttcgttgtctat	Nos CalFluor590_16
gtttccctttcacagaaaca	Nos CalFluor590_17
gccacgacgattgaacaagt	Nos CalFluor590_18
tccattcatcaactttcgga	Nos CalFluor590_19
aaatgaaggcgaccagttgc	Nos CalFluor590_20
cgaaatttttcggccgcaag	Nos CalFluor590_21
ttcaaagtgttcctttttca	Nos CalFluor590_22
tgatacgattgacagttcga	Nos CalFluor590_23
cttgctatttcctttagcaa	Nos CalFluor590_24
acaatgaatgcgtagccgac	Nos CalFluor590_25
tactcttcgcttatctatca	Nos CalFluor590_26
gcggttgtttcatgtgtgaa	Nos CalFluor590_27
cgagccattgaatttttcat	Nos CalFluor590_28
aaccatttctttatttggca	Nos CalFluor590_29
tccaagttgctgcggaacat	Nos CalFluor590_30
tcctctggcgtgaaaagcag	Nos CalFluor590_31
tgcaggcccagaatgttgag	Nos CalFluor590_32
ccactggtatccaaatacat	Nos CalFluor590_33
gtaatgggcggactcaaagt	Nos CalFluor590_34
tcggccagaaaagggaagtg	Nos CalFluor590_35
cataaggagcgaattggcgg	Nos CalFluor590_36
caagtggtagtggtactgtc	Nos CalFluor590_37
ttgctggtgactcgcactag	Nos CalFluor590_38
aaggatcgcgcaatctcgtc	Nos CalFluor590_39
cgtcacctgcgcaaagattt	Nos CalFluor590_40
catagccattggtcgcgaac	Nos CalFluor590_41
taggacatgcgaccgagatc	Nos CalFluor590_42
cattaagttgccgccattgg	Nos CalFluor590_43
agtgggtggcgagtggaatg	Nos CalFluor590_44
cacacgttgttcagatgctc	Nos CalFluor590_45
ggctggtatatacgacatgt	Nos CalFluor590_46
ctgcaaacccattgtattgg	Nos CalFluor590_47
cgagattggtggacacagtg	Nos CalFluor590_48

Supplementary Table 6

pgc smFISH probes

Probe Sequence (5' to 3')	Probe name
agtactccatctggtagtcg	Pgc 24 CalFluor590_1
gcaggagctgtcttcaaaaa	Pgc 24 CalFluor590_2
taggatgccatcgaggcatc	Pgc 24 CalFluor590_3
catggactcgaatccattgt	Pgc 24 CalFluor590_4
aactcctcgcgcacttgatg	Pgc 24 CalFluor590_5
tggcagagctcattcatctc	Pgc 24 CalFluor590_6
gctcaagttttgctggaaaa	Pgc 24 CalFluor590_7
tctatccgcgatgaccggcg	Pgc 24 CalFluor590_8
ggaggtccagtcagaatctc	Pgc 24 CalFluor590_9
tatcacaataagttggcttt	Pgc 24 CalFluor590_10
gcaaacgaactgctaaaact	Pgc 24 CalFluor590_11
ttcacgatgttccactcatg	Pgc 24 CalFluor590_12
accgagcacttatcaaaagt	Pgc 24 CalFluor590_13
ctgaaggctggtagttacaa	Pgc 24 CalFluor590_14
gaaactatgcatacgatcgc	Pgc 24 CalFluor590_15
tacacggacaaattgacttc	Pgc 24 CalFluor590_16
ttcacgaaagcaaacatttg	Pgc 24 CalFluor590_17
agagtgacaaaacaatgcga	Pgc 24 CalFluor590_18
ccatatgcgattgattggta	Pgc 24 CalFluor590_19
gccatttttagatctaggac	Pgc 24 CalFluor590_20
gcaataccgaaggcaaatta	Pgc 24 CalFluor590_21
cttgttcttgaatacattag	Pgc 24 CalFluor590_22
tgagaaatttcgagcttccc	Pgc 24 CalFluor590_23
aacgattgcgaatcgaaaat	Pgc 24 CalFluor590_24

Supplementary Table 7

gcl smFISH probes

Probe Sequence (5' to 3')	Probe name
atggatcccactatttgacc	gcl 48 CalFluor590_1
cacctcagcgacgttcatat	gcl 48 CalFluor590_2
cccaaagatgaatcagtgct	gcl 48 CalFluor590_3
gcgccttgtatatgtactgg	gcl 48 CalFluor590_4
ccgagttcttttcctccttg	gcl 48 CalFluor590_5
cttatccagagccatgacag	gcl 48 CalFluor590_6
tagaagtatggactctggct	gcl 48 CalFluor590_7
ccacgtcccattgaacatag	gcl 48 CalFluor590_8
atgaaattctgctgggcttc	gcl 48 CalFluor590_9
gtcgtccaggattgtgatct	gcl 48 CalFluor590_10
tacatggaaccaaagacagc	gcl 48 CalFluor590_11
gctgactcgatttcgatctc	gcl 48 CalFluor590_12
ggccaataccgagataacat	gcl 48 CalFluor590_13
tctaagtggaacaacgtggc	gcl 48 CalFluor590_14
ggcacacttgtcgatgattc	gcl 48 CalFluor590_15
ccggactaatgttatccacc	gcl 48 CalFluor590_16
cggcctcgtaatactggata	gcl 48 CalFluor590_17
actgaaaggtggacttcttg	gcl 48 CalFluor590_18
tgcttagcagattgatctgg	gcl 48 CalFluor590_19
caggttcggttgcttactat	gcl 48 CalFluor590_20
cactcatcagctcaatggag	gcl 48 CalFluor590_21
gcatcacatacaaatcggga	gcl 48 CalFluor590_22
cagtagagtgtacagcgaga	gcl 48 CalFluor590_23
gtcgtaggaacatccatgtg	gcl 48 CalFluor590_24
ctctggatcgtagtcgggat	gcl 48 CalFluor590_25
aaaagctcctgcgtctttag	gcl 48 CalFluor590_26
ctggacgacatcaccactag	gcl 48 CalFluor590_27
agcggctggtaaagtatgtc	gcl 48 CalFluor590_28
aagaccttcacataaggctg	gcl 48 CalFluor590_29
gtactgggttcgtagcttct	gcl 48 CalFluor590_30
gatccatgtagtggttggtc	gcl 48 CalFluor590_31
ttgtgaatgtgccgatacag	gcl 48 CalFluor590_32
acgtagtaatgcatcccagt	gcl 48 CalFluor590_33
ctgaggactacaatcctcct	gcl 48 CalFluor590_34
ctcaaagaactgctcatcgt	gcl 48 CalFluor590_35
aggctcaagcaacatacgtc	gcl 48 CalFluor590_36
gagatccatgccaaagttga	gcl 48 CalFluor590_37
gaatgttcagtctacgcgag	gcl 48 CalFluor590_38
atgaacttgcgtttcgtctg	gcl 48 CalFluor590_39
gtttatcgaggtcaccgtgg	gcl 48 CalFluor590_40
gaacagatctcggacgtttg	gcl 48 CalFluor590_41
ctcgttcttttcgaggctaa	gcl 48 CalFluor590_42
ctttgggtctaacaccatga	gcl 48 CalFluor590_43
aaatgagaagtggatgcacc	gcl 48 CalFluor590_44
ctctgattcggtggcattac	gcl 48 CalFluor590_45
gagcggaacaatttccttga	gcl 48 CalFluor590_46
ccgatttccgaaatgggtat	gcl 48 CalFluor590_47
gctcagagtcaccaatgaaa	gcl 48 CalFluor590_48

Supplementary Table 8

ccr4 smFISH probes

Probe Sequence (5' to 3')	Probe name
gcagagcacattatagcaca	ccr4 CalFluor 590_1
cagtatccgtacatttgtcg	ccr4 CalFluor 590_2
acttttttcggtactcccag	ccr4 CalFluor 590_3
tgccgtatctcgtcgataat	ccr4 CalFluor 590_4
ctgcagactgataatgtccg	ccr4 CalFluor 590_5
tagaattgctccgtttcgat	ccr4 CalFluor 590_6
tgagttccggcaggaagaag	ccr4 CalFluor 590_7
gacttcggtgagaagattcc	ccr4 CalFluor 590_8
cagctcggacatagtcttag	ccr4 CalFluor 590_9
cacagccatcgacgtacttc	ccr4 CalFluor 590_10
ttggacgccctgaagaatat	ccr4 CalFluor 590_11
aatgattccttgatcagcgt	ccr4 CalFluor 590_12
attgccagctgattgaactc	ccr4 CalFluor 590_13
attacgcggttcagcatgtt	ccr4 CalFluor 590_14
cagaccgatgttatccttag	ccr4 CalFluor 590_15
cttgacgtcgcagaactcag	ccr4 CalFluor 590_16
ctaagcatcatcgtctggat	ccr4 CalFluor 590_17
tcgtcgatgatcgtcttcaa	ccr4 CalFluor 590_18
tgaccaggtcggaaactgtg	ccr4 CalFluor 590_19
tggacagcattggagtcgtt	ccr4 CalFluor 590_20
gaatcgggtagcgagttgaa	ccr4 CalFluor 590_21
ttgccgagaaactccacaac	ccr4 CalFluor 590_22
ccttgaagtccaaatgatcc	ccr4 CalFluor 590_23
aaactcgttggtgtcgttcg	ccr4 CalFluor 590_24
gaggctagcttgaacgagtg	ccr4 CalFluor 590_25
cattatgtcctcgttgtagg	ccr4 CalFluor 590_26
cgaacgtatagttggtgtgc	ccr4 CalFluor 590_27
tagtcgatgatgcccttaaa	ccr4 CalFluor 590_28
gcccgtcttcgtgtagaaaa	ccr4 CalFluor 590_29
aatcattggagacaggaccc	ccr4 CalFluor 590_30
acaaccttattctcgcgcag	ccr4 CalFluor 590_31
tatatgcggatgtgggcatc	ccr4 CalFluor 590_32
agcagtgggaagtgatcaga	ccr4 CalFluor 590_33
tatgcatcagctctagctcg	ccr4 CalFluor 590_34
attgatcagcccgttaggag	ccr4 CalFluor 590_35
cgcgtctattctattgctac	ccr4 CalFluor 590_36
tactggttgtggtgctgttg	ccr4 CalFluor 590_37
agacgatggcatccgctatt	ccr4 CalFluor 590_38
gaggattcactgcttttctt	ccr4 CalFluor 590_39
attttgatgcgagcagatcg	ccr4 CalFluor 590_40
agggaccggattagaaatca	ccr4 CalFluor 590_41
ggggatggtgggtacaagag	ccr4 CalFluor 590_42
cacaacgtcgtcagcttata	ccr4 CalFluor 590_43
ttgcgatccagcgcacaaag	ccr4 CalFluor 590_44
aatgtgttgcattggtgagc	ccr4 CalFluor 590_45
ccttcctattacgattacga	ccr4 CalFluor 590_46
cgtaagaggtgtgcagcaag	ccr4 CalFluor 590_47
cgatggtgaaaacgtgcagt	ccr4 CalFluor 590_48

Supplementary Table 9

osk smFISH probes

Probe Sequence (5' to 3')	Probe name
cgcgcacggatatatagttg	Osk CalFluor 590_1
cacctcactatctatatcgg	Osk CalFluor 590_2
atatcgtgattccattctgg	Osk CalFluor 590_3
cgatattcactcttgatgct	Osk CalFluor 590_4
tgcagtggaaatggattgcc	Osk CalFluor 590_5
caggaaatccgtcacgttgt	Osk CalFluor 590_6
cagcggtcacattgggaatg	Osk CalFluor 590_7
tcgctttcaggttgaagatc	Osk CalFluor 590_8
aggaggtgaccgttcttcag	Osk CalFluor 590_9
tttctggttgagcaccatat	Osk CalFluor 590_10
tatgttctccagggacggag	Osk CalFluor 590_11
gtttgaagggattcttccag	Osk CalFluor 590_12
ggtgttcagctgggacagag	Osk CalFluor 590_13
cttttcatccgttatcttgg	Osk CalFluor 590_14
catttgatgcagcgaaaccg	Osk CalFluor 590_15
agatgcttccaattatcctg	Osk CalFluor 590_16
ggcttgctggtagaaattgt	Osk CalFluor 590_17
gcatttttggcgcatttacg	Osk CalFluor 590_18
gctgtagatgttgatgggta	Osk CalFluor 590_19
caggtggagccaaattgatt	Osk CalFluor 590_20
ttttctggctttgggttctg	Osk CalFluor 590_21
caaatggcggttttcagtcg	Osk CalFluor 590_22
gccgttaaatggatgcacaa	Osk CalFluor 590_23
tggcgtctcttcattatgtt	Osk CalFluor 590_24
gttaaaatcgttggcgtggg	Osk CalFluor 590_25
agaatcgttgtaggttccac	Osk CalFluor 590_26
aatccgagttaatcgtcagc	Osk CalFluor 590_27
aagtccagcagataggcatc	Osk CalFluor 590_28
aaaatcatcgcccataagcg	Osk CalFluor 590_29
tccattcgggcgagatatag	Osk CalFluor 590_30
acgcctaaatcggcatttta	Osk CalFluor 590_31
caaagtcctgactgcaggac	Osk CalFluor 590_32
attgatcgtcagtccggata	Osk CalFluor 590_33
tgagtaccctcgggtaattg	Osk CalFluor 590_34
ccgatccgatattgacgatg	Osk CalFluor 590_35
gatctgaaccaaaggcttgc	Osk CalFluor 590_36
tcagtagccgaaaatcgtgc	Osk CalFluor 590_37
accaatcgcatattgtgcat	Osk CalFluor 590_38
gcggtgcaagatttgttaga	Osk CalFluor 590_39
ttatcgtgacaatagttgcc	Osk CalFluor 590_40
atcggtagattttgtcacat	Osk CalFluor 590_41
gcattcgcttcggataaact	Osk CalFluor 590_42
atgtcgatgacctttaggtg	Osk CalFluor 590_43
cctttcgttgattagacagg	Osk CalFluor 590_44
agcaatcaaatcgcaccacg	Osk CalFluor 590_45
gatagggttccttggaaccg	Osk CalFluor 590_46
cgaccgattttgttccagaa	Osk CalFluor 590_47
caataacttgcagtacgcgc	Osk CalFluor 590_48

Supplementary Table 10

Red *gcl* smFISH probes

Probe Sequence (5' to 3')	Probe name
tccaaatcttttagttatgg	Gcl red Cal Fl 590_1
ttccgttcgctgtcaatttt	Gcl red Cal Fl 590_2
aattctacgcgagtctgatt	Gcl red Cal Fl 590_3
cgttcactggcttcgtttca	Gcl red Cal Fl 590_4
gagcgcaggaattcttactt	Gcl red Cal Fl 590_5
cagcgacgttcatatgcatg	Gcl red Cal Fl 590_6
cccaaagatgaatcagtgct	Gcl red Cal Fl 590_7
ccgagttcttttcctccttg	Gcl red Cal Fl 590_8
gtacaccttatgcaggtgcc	Gcl red Cal Fl 590_9
ccacgtcccattgaacatag	Gcl red Cal Fl 590_10
gtcgtccaggattgtgatct	Gcl red Cal Fl 590_11
tacatggaaccaaagacagc	Gcl red Cal Fl 590_12
ggccaataccgagataacat	Gcl red Cal Fl 590_13
ggcacacttgtcgatgattc	Gcl red Cal Fl 590_14
cggcctcgtaatactggata	Gcl red Cal Fl 590_15
attgatctggaaccactgaa	Gcl red Cal Fl 590_16
gatgtgcctaagcaggttcg	Gcl red Cal Fl 590_17
tacaaatcgggactggcggt	Gcl red Cal Fl 590_18
atgtgcgcagtagagtgtac	Gcl red Cal Fl 590_19
cgggtcctctggatcgtagt	Gcl red Cal Fl 590_20
ctggacgacatcaccactag	Gcl red Cal Fl 590_21
gccaagaacgagcgttcctc	Gcl red Cal Fl 590_22
gtactgggttcgtagcttct	Gcl red Cal Fl 590_23
ccttgggtataatattgtca	Gcl red Cal Fl 590_24
acgtagtaatgcatcccagt	Gcl red Cal Fl 590_25
aactgctcatcgtccagctg	Gcl red Cal Fl 590_26
caggctcaagcaacatacgt	Gcl red Cal Fl 590_27
atccatgccaaagttgaagc	Gcl red Cal Fl 590_28
gtgccgacgaatgttcagtc	Gcl red Cal Fl 590_29
atgaacttgcgtttcgtctg	Gcl red Cal Fl 590_30
gaacagatctcggacgtttg	Gcl red Cal Fl 590_31
tctaacaccatgagtggcac	Gcl red Cal Fl 590_32
accaccagcatgtttattga	Gcl red Cal Fl 590_33
ctcgctgagcggaacaattt	Gcl red Cal Fl 590_34
cagcggtctgtcgctgtttg	Gcl red Cal Fl 590_35
gctcagagtcaccaatgaaa	Gcl red Cal Fl 590_36
cgccagttgtccgcagattg	Gcl red Cal Fl 590_37
ctgcgggtaaatgaacaaaa	Gcl red Cal Fl 590_38
gcgatggcgataaacgaatt	Gcl red Cal Fl 590_39
tcgatgtacaaacgtctaga	Gcl red Cal Fl 590_40
atatagatctgcgcattcca	Gcl red Cal Fl 590_41
ccatttaagtagatgacgta	Gcl red Cal Fl 590_42
ttgcaacgcctacatattgt	Gcl red Cal Fl 590_43
aacttgcaggattcgaagac	Gcl red Cal Fl 590_44
ctgtgcgtacatttagcgag	Gcl red Cal Fl 590_45

Supplementary Table 11

Green *gcl* smFISH probes

Probe Sequence (5' to 3')	Probe name
ctctgttttgctgcagttgg	Gcl green Quasar670_1
catgcggtttaagccatttc	Gcl green Quasar670_2
aaataaaataaacacgggct	Gcl green Quasar670_3
cagttacagtgccgtacttt	Gcl green Quasar670_4
tcccactatttgacccatgg	Gcl green Quasar670_5
cttccgtcgattgctgaaca	Gcl green Quasar670_6
gcgccttgtatatgtactgg	Gcl green Quasar670_7
cttatccagagccatgacag	Gcl green Quasar670_8
tagaagtatggactctggct	Gcl green Quasar670_9
atgaaattctgctgggcttc	Gcl green Quasar670_10
ccaggctagcaacggtgatc	Gcl green Quasar670_11
gctgactcgatttcgatctc	Gcl green Quasar670_12
tctaagtggaacaacgtggc	Gcl green Quasar670_13
ccggactaatgttatccacc	Gcl green Quasar670_14
gtggacttcttgactccgac	Gcl green Quasar670_15
gcttactataaatgcttagc	Gcl green Quasar670_16
gggcactcatcagctcaatg	Gcl green Quasar670_17
cgagaactccgtctgcatca	Gcl green Quasar670_18
ggatgcagtcgtaggaacat	Gcl green Quasar670_19
aaaagctcctgcgtctttag	Gcl green Quasar670_20
agcggctggtaaagtatgtc	Gcl green Quasar670_21
aagaccttcacataaggctg	Gcl green Quasar670_22
gatccatgtagtggttggtc	Gcl green Quasar670_23
ttgtgaatgtgccgatacag	Gcl green Quasar670_24
gaggactacaatcctcctgc	Gcl green Quasar670_25
gcatcgcatgcagttctcaa	Gcl green Quasar670_26
gtccagcgccacttctggta	Gcl green Quasar670_27
gcgagtccatgatcaggatg	Gcl green Quasar670_28
taagcactcgctcgtgctcg	Gcl green Quasar670_29
gtttatcgaggtcaccgtgg	Gcl green Quasar670_30
ctcgttcttttcgaggctaa	Gcl green Quasar670_31
gaagtggatgcaccaacttt	Gcl green Quasar670_32
ttgaaactctgattcggtgg	Gcl green Quasar670_33
ccgatttccgaaatgggtat	Gcl green Quasar670_34
gaatcatcagcactggacgg	Gcl green Quasar670_35
ctagccgaccaagcgattct	Gcl green Quasar670_36
attggtttggtggactgctc	Gcl green Quasar670_37
aaacaactacaacgagcccg	Gcl green Quasar670_38
gagtgatcagccgtagctaa	Gcl green Quasar670_39
acgattgcggatatgcttat	Gcl green Quasar670_40
gcgtcattacatgatactag	Gcl green Quasar670_41
ggcactagagtgtgcgtcta	Gcl green Quasar670_42
atttctttgccgcgtttttt	Gcl green Quasar670_43
gggacagtaattacatgcgt	Gcl green Quasar670_44
tgatcactcaaggtaatagt	Gcl green Quasar670_45

Supplementary Table 12

Alexa488 *pgc* smFISH probes

Probe Sequence (5' to 3')	Probe name
catctggtagtcgcacattt	pgc Alexa 488 1
aatccattgtcgtaggatgc	pgc Alexa 488 2
ttcacgatgttccactcatg	pgc Alexa 488 3
aaactatgcatacgatcgcc	pgc Alexa 488 4
acacggacaaattgacttca	pgc Alexa 488 5
agagtgacaaaacaatgcga	pgc Alexa 488 6
ggacaaccatatgcgattga	pgc Alexa 488 7
tgagaaatttcgagcttccc	pgc Alexa 488 8
gcaggagctgtcttcaaaaa	pgc Alexa 488 9
aactcctcgcgcacttgatg	pgc Alexa 488 10
tggcagagctcattcatctc	pgc Alexa 488 11
gctcaagttttgctggaaaa	pgc Alexa 488 12
tctatccgcgatgaccggcg	pgc Alexa 488 13
ggaggtccagtcagaatctc	pgc Alexa 488 14
tatcacaataagttggcttt	pgc Alexa 488 15
gcaaacgaactgctaaaact	pgc Alexa 488 16
cttgttcttgaatacattag	pgc Alexa 488 17

Supplementary Table 13

SUPPLEMENTARY REFERENCES

1. Bolte, S. & Cordelieres, F.P. A guided tour into subcellular colocalization analysis in light microscopy. *Journal of microscopy* **224**, 213-232 (2006).
2. Markow, T.A., Beall, S. & Matzkin, L.M. Egg size, embryonic development time and ovoviviparity in Drosophila species. *Journal of evolutionary biology* **22**, 430-434 (2009).
3. mod, E.C. *et al.* Identification of functional elements and regulatory circuits by Drosophila modENCODE. *Science* **330**, 1787-1797 (2010).
